# The Regulation of Vasomotor and Cardiorespiratory Pulsations Is Disrupted in Primary Central Nervous System Lymphoma: A Case–Control fMRI Study

**DOI:** 10.1002/hbm.70495

**Published:** 2026-03-11

**Authors:** Valter Poltojainen, Matti Järvelä, Nina Keinänen, Michaela K. Bode, Juha‐Matti Isokangas, Hanne Kuitunen, Juha Nikkinen, Vesa Korhonen, Niko Huotari, Lauri Raitamaa, Janne Kananen, Heta Helakari, Tommi Kalevi Korhonen, Sami Tetri, Outi Kuittinen, Vesa Kiviniemi

**Affiliations:** ^1^ Oulu Functional NeuroImaging‐(OFNI), Research Unit of Health Sciences and Technology University of Oulu Oulu Finland; ^2^ Medical Imaging, Physics and Technology (MIPT), Faculty of Medicine University of Oulu Oulu Finland; ^3^ Medical Research Center (MRC) Oulu University Hospital Oulu Finland; ^4^ Anesthesiology, Oulu University Hospital Oulu Finland; ^5^ Department of Diagnostic Radiology, Oulu University Hospital, Research Unit of Health Sciences and Technology (HST) University of Oulu Oulu Finland; ^6^ Oncology and Hematology Oulu University Hospital Oulu Finland; ^7^ Oncology and Radiotherapy Oulu University Hospital Oulu Finland; ^8^ Clinical Neurophysiology Oulu University Hospital Oulu Finland; ^9^ Neurosurgery, Clinical Neuroscience University of Oulu/Oulu University Hospital Oulu Finland; ^10^ Cancer Center Kuopio University Hospital Kuopio Finland; ^11^ Faculty of Health Medicine, Institute of Clinical Medicine University of Eastern Finland Kuopio Finland

**Keywords:** lymphoma, malignancy, physiology, pulsation

## Abstract

Primary central nervous system lymphoma (PCNSL) alters (peri)vascular structures while increasing vasomotor and cardiorespiratory pulsations within the brain. Vasomotor pulsations may arise from amplitude modulations of respiratory (RPE) and cardiovascular (CHE) pulsations while cardiovascular fluctuations may be modulated by respiration through cardiorespiratory amplitude modulation (CREM). In this study, we examined glymphatic cerebrospinal fluid convection in brains of PCNSL patients by assessing these waves. Thirty PCNSL patients (median 66y; 9 females) and 40 healthy age‐matched controls (median 62y; 29 females) were scanned using an fMRI‐based MREG_BOLD_ sequence. Respective MREG_BOLD_ fluctuation amplitudes (AF_RPE_; AF_CHE_; AF_CREM_) were compared between groups using nonparametric permutation. Regional amplitudes were compared using Mann–Whitney analysis and Cox survival analysis. Subject‐specific pulsations were analyzed through Z‐score mapping. AF_CREM_ and AF_RPE_ were significantly elevated across PCNSL brains, with lesser increases in AF_CHE_. However, only significant increases in AF_RPE_ remained after correcting for sex and head displacement. AF_RPE_ showed a link to mortality as it was markedly elevated in deceased patients. While elevations in all pulsations were present within (peri)tumoral regions, AF_RPE_ elevations extended into extra‐tumoral white matter and grey matter. Thus, altered cardiorespiratory fluctuations give rise to dysfunctional vasomotor and CSF pulsations in PCNSL, predicting impaired glymphatic function.

## Highlights

- Patients with PCNSL showed increased amplitudes of low‐frequency respiratory (AF_RPE_) and cardiovascular (AF_CHE_) modulation and cardiorespiratory amplitude modulation (AF_CREM_) in brain.
- Increased AF_RPE_ in PCNSL shows a link with mortality during follow‐up.

## Graphical abstract

Using fast functional MRI, we assessed low‐frequency oscillations arising from cardiorespiratory pulsations and oscillatory interaction between respiratory and cardiac pulsations in primary central nervous system lymphoma. Respiratory‐related oscillations showed brain‐wide increases with a link to mortality whereas increases in cardiovascular‐related oscillations were localized more prominently to macroscopically visible tumor areas.

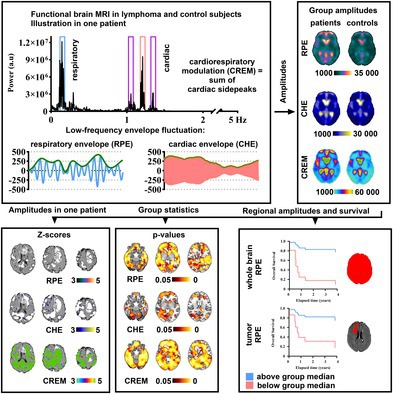

**Abbreviations**
BBB: blood–brain barrier
BOLD: blood‐oxygenation‐level‐dependent
CBF: cerebral blood flow
CBV: cerebral blood volume
CHE: cardiovascular pulse envelope modulation
CMRO_2_
: cerebral metabolic rate of oxygen
CREM: cardiorespiratory envelope modulation
CSF: cerebrospinal fluid
CVR: cerebrovascular reactivity
FLAIR: fluid attenuated inversion recovery sequence
GM: grey matter
MREG_BOLD_
: magnetic resonance encephalography
PCNSL: primary central nervous system lymphoma
*p*CO_2_
: partial pressure of CO_2_
ROI: region of interest
RPE: respiratory pulse envelope modulation
VLF: very‐low frequency
WM: white matter

## 1 Introduction

Primary central nervous system lymphomas (PCNSL) are a rare type of non‐Hodgkin lymphoma. Some 50 new cases are reported in Finland annually, representing one of the highest reported incidences in the world, with onset peaking among those aged 60–70 years (Puhakka et al. 2022). In histological examinations, PCNSL infiltration is noted as an increase of perivascular lymphocytes that organize as cuff‐like structures surrounding the cerebral blood vessels (Aho et al. 1993; Molnár et al. 1999; Ambady et al. 2017). Due to tumor infiltration, there is enlargement of perivascular spaces, retraction of astrocyte end‐feet, variability in vessel wall structure, aberrant patterns of vascularization, and finally disruption of the blood–brain barrier (BBB) (Aho et al. 1993; Molnár et al. 1999; Ambady et al. 2017; Ribatti et al. 2023).

Upon diagnostic MRI, PCNSL typically presents with a homogenously enhancing mass or masses localized within the periventricular white matter (Grommes and DeAngelis 2017; Poltojainen et al. 2022). However, since lymphocytes may infiltrate behind a seemingly intact BBB, structural neuroimaging methods may overlook diseased foci that could act as seeding regions for relapse or disease progression (Ambady et al. 2017; Poltojainen et al. 2022). The rapid and diffuse infiltration pattern of PCNSL across the whole brain proceeds to aggressive disease progression, with only 50% survival at 1 year after diagnosis (Puhakka et al. 2022), which has motivated increasing efforts to find promising chemotherapeutic regimens in recent decades. For example, increasing the BBB permeability to chemotherapeutics by intra‐arterial mannitol infusion has shown promise (Angelov et al. 2009; Kuitunen et al. 2017), insofar as the resulting increased cerebrospinal fluid (CSF) convection may improve drug pharmacokinetics and promote patient survival (Shah et al. 2007).

Considering that PCNSL infiltrates within perivascular spaces that are important routes for CSF trafficking and that PCNSL has marked effects on the cerebral vasculature, we have previously sought to establish the effects of this disorder on the brain pulsations that drive vasomotion and CSF convection within the brain. To this end, we have deployed an fMRI‐based magnetic resonance encephalography (MREG_BOLD_) technique that is inherently sensitive to fluid flow and vessel motion and enables precise, unaliased extraction of cardiorespiratory pulsations which may be perturbed in various disease states (Kiviniemi et al. 2016; Huotari et al. 2019; Kananen et al. 2020; Tuovinen et al. 2020; Raitamaa et al. 2021; Rajna et al. 2021; Helakari et al. 2022; Järvelä et al. 2022; Tuunanen et al. 2024; Poltojainen et al. 2025).

Previously, we found brain‐wide increases in total MREG_BOLD_ signal variability, which was predictive of incipient mortality in patients suffering from PCNSL (Poltojainen et al. 2022). In subsequent investigations, we discovered brain‐wide increases in vasomotor (< 0.1 Hz) pulsation with lesser increases in respiratory (0.25 Hz) and cardiovascular (1 Hz) pulsations while increased vasomotor pulsation was linked with mortality in PCNSL patients (Poltojainen et al. 2025). We suspected that our findings of increased pulsation amplitudes in PCNSL may be linked to altered pulsation of the vessels themselves, as well as altered cerebral blood flow (CBF), cerebral blood volume (CBV), and CSF flow patterns.

Traditionally, the low‐frequency (< 0.1 Hz) blood‐oxygenation‐level dependent (BOLD) fMRI signal has served to capture changes in brain activity within functionally connected brain areas (Ogawa et al. 1992; Biswal et al. 1995; Bandettini et al. 1992). However, a considerable component of this signal necessarily reflects non‐neural sources arising from the vasculature itself. Among these sources are the CBF and CBV fluctuations arising from breathing (Wise et al. 2004) and cardiovascular functions (Shmueli et al. 2007; Whittaker et al. 2019) that have been modelled using externally recorded peripheral signals. Additionally, vascular function is influenced by autoregulation mechanisms that regionally modulate vasomotion (Whittaker et al. 2019; Willie et al. 2014; Katura et al. 2006; Sliwka et al. 2001; Obrig et al. 2000). More recently, we have developed methods to extract the low‐frequency amplitude of fluctuations (AF) of intracranial respiratory pulse envelope (AF_RPE_) and cardiovascular pulse envelope (AF_CHE_) directly from the unaliased MREG_BOLD_ signal (Tuunanen et al. 2024; Huotari et al. 2022). The AF_RPE_ models low‐frequency amplitude modulations in the respiratory (0.25 Hz) pulsations that drive the (peri)venous fluid flow within the brain (Santisakultarm et al. 2012; Dreha‐Kulaczewski et al. 2015; Lloyd et al. 2020; Söderström et al. 2025) whereas AF_CHE_ models low‐frequency amplitude modulations in the cardiovascular (1 Hz) pulsations that drive the (peri)arterial fluid flow within the brain (Mitchell et al. 2011; Posse et al. 2013; Dagli et al. 1999; Bhattacharyya and Lowe 2004).

Additionally, the direct physical interactions of intracranial respiratory and cardiovascular fluctuations have previously been detected with phase‐contrast MRI and echo planar fMRI methods that characterized how respiration modulates the systolic flow speed of CSF and blood (Lloyd et al. 2020; Klose et al. 2000; Friese et al. 2004; Brooks et al. 2008). More recently, we used MREG_BOLD_ signals to describe how respiratory fluctuations modulate the intracerebral amplitude of cardiac fluctuations in a process called cardiorespiratory envelope modulation (CREM) (Raitamaa et al. 2021; Raitamaa et al. 2024). The amplitude of this modulation (AF_CREM_) manifests in MREG_BOLD_ frequency spectra and has distinctive spatiotemporal behavior that stands in contrast to ordinary respiratory and cardiovascular fluctuations and may bear relation to CSF convection.

In the present study, we first tested the hypothesis that our previous finding of brain‐wide increases in vasomotor MREG_BOLD_ fluctuation amplitude in PCNSL patients may arise from increased AF_RPE_ and/or AF_CHE_. We next tested the hypothesis that the intracerebral AF_CREM_ is altered in PCNSL patients. To test these joint hypotheses, we calculated AF_RPE_, AF_CHE_, and AF_CREM_ directly from critically sampled MREG_BOLD_ data in freely breathing, awake PCNSL patients and in healthy control subjects.

## 2 Materials and Methods

This study adheres to the Declaration of Helsinki and was conducted after obtaining institutional approval by the Ethical Committee of Pohde Wellbeing Services County of North Ostrobothnia (formerly known as the Northern Ostrobothnia Hospital District of Oulu University Hospital) (permits 53/2012, 274/2020, 181/2023, Fimea 2023/005604). Written informed consent was obtained from all subjects.

In this retrospective case–control study, our final analysis included 30 confirmed PCNSL patients (median age 66 years, nine females) and 40 healthy age‐matched controls (median age 62 years, 29 females). All MREG_BOLD_ scans were conducted at resting state without specific breathing or other tasks. The general inclusion criteria for the control group were absence of neurological disease and normal findings in brain structural MRI (assessed by neuroradiologist VK). We excluded from analysis any individuals with mean head motion exceeding the isotropic 3 mm voxel size, as defined by MCFLIRT reports. There were some instances of non‐neurological comorbidities among the patient and control groups. For complete demographics, see Table 1.

**TABLE 1:** Demographics.

Parameter	Controls (*n* = 40)	PCNSL (*n* = 30)	Significance (*p*)
Age (years)	62 [61–66]	66 [60–71]	0.063 (ns)
Females (*n*)	29 (73%)	9 (30%)	0.0006 (***)
Relative motion (mm)	0.035 [0.029–0.044]	0.036 [0.032–0.044]	0.35 (ns)
Absolute motion (mm)	0.161 [0.113–0.219]	0.191 [0.149–0.323]	0.022 (*)
Framewise motion (mm)	0.061 [0.051–0.077]	0.067 [0.058–0.079]	0.21 (ns)
Respiratory rate (Hz)	0.25 [0.21–0.29]	0.25 [0.21–0.33]	0.51 (ns)
Cardiac rate (Hz)	1.12 [1.03–1.25]	1.19 [1.00–1.30]	0.71 (ns)
Comorbidity (*n*)			
Arrhythmia	1 (2.5%)	5 (17%)	
Asthma or sleep apnea	4 (10%)	2 (7%)	
Coronary artery disease	0 (0%)	3 (10%)	
Hypercholesterolemia	5 (13%)	5 (17%)	
Hypertension	7 (18%)	8 (27%)	
Hypothyroidism	2 (5%)	3 (10%)	
Psychiatric	0 (0%)	4 (13%)	
Deceased (*n*)	0 (0%)	7 (23%)	
Method for diagnosis (*n*)			
Resection		3 (10%)	
Needle biopsy		17 (56%)	
CSF aspiration		2 (7%)	
Unsolved due to referral		8 (27%)	
Diagnosis (*n*)			
DLBCL		29 (97%)	
Lymphocytic lymphoma		1 (3%)	
MREG scanning phase			
Before 1st treatment		22 (73%)	
Before 2nd treatment		4 (13%)	
Before 3rd treatment		3 (10%)	
Before 4th treatment		1 (3%)	
Seizures (*n*)		12 (40%)	

*Note:* We present median values and corresponding interquartile range (IQR) limits, or number and corresponding %‐portion. Statistical *p*‐values represent results from an exact two‐tailed Mann–Whitney U‐test for continuous data, or an exact two‐tailed Fisher's test for categorical data. The asterisk (*) denotes significant *p*‐values; ns = non‐significant difference. DLBC = Diffuse Large B‐Cell Lymphoma. Note that some patients had been referred from other institutions after their pathologically confirmed diagnosis (stereotactic needle biopsy or resection), and due to restrictions regarding patient records, we could not ascertain the exact method for diagnosis in these cases.

Most of our present PCNSL cases (*n* = 21; 70%) and control subjects (*n* = 30; 75%) had been recruited for our previous study where we had addressed the total signal variation of MREG_BOLD_ signal (Poltojainen et al. 2022), and all participants had been included in a more recent study where we had addressed the vasomotor, respiratory, and cardiovascular pulsation amplitudes (Poltojainen et al. 2025). For the present study, we had consecutively enrolled 45 cases suspected of PCNSL in initial radiological assessment or with certain diagnosis between 4/2019 and 9/2021 and assessed clinical outcome during 8/2024. After initial assessment for their eligibility and inclusion, we had retrospectively obtained a final diagnosis through stereotactic needle biopsy, tumor resection, or cerebrospinal fluid aspiration. Clinical evaluation also included excluding systemic lymphoma in all patients, excluding testicular lymphoma in male patients, and evaluation for concomitant ocular involvement. We excluded one PCNSL patient due to excessive supra‐voxel‐scale head displacement, and 14 suspected cases due to discordant histopathological diagnosis such as glioma.

Most of the PCNSL patients in the present study (*n* = 27; 90%) had received induction chemotherapy with intra‐arterial mannitol infusion to induce a transient BBB‐disruption (BBBD), aiming to enhance intraparenchymal concentrations of chemotherapeutics in pursuit of curative treatment (Angelov et al. 2009; Kuitunen et al. 2017). In this procedure, patients are first given a cycle of cytoreductive MATRix treatment consisting of cytarabine, methotrexate, rituximab, and thiotepa. The actual chemotherapy regimen with BBBD‐augmentation started some 4 weeks later and consisted of rituximab, methotrexate, carboplatin, cyclophosphamide, and etoposide. Consolidation therapy consisted of high‐dose carmustine‐thiotepa conditioning chemotherapy and autologous stem cell transplantation.

We acquired MREG_BOLD_ imaging in the PCNSL cases at the earliest possible treatment phase; before the first treatment in 22 patients (73%), before the second treatment in four (13%), before the third treatment in three (10%), and before the fourth treatment in one case (3%). Seven of the 30 PCNSL patients (23%) died between 4/2019 and 8/2024, reflecting better overall survival than the 53% average 5‐year mortality rate in previous cohorts with BBBD‐augmented treatment (Angelov et al. 2009; Kuitunen et al. 2017). One of these deceased patients was excluded from BBBD—treatment due to rapid disease progression. Four had received first‐line BBBD‐treatment; one had disease progression despite BBBD‐treatment and was offered palliative whole‐brain radiation therapy (WBRT); one achieved curative response, but had relapsed and died of rapid progression some 2 years later; one achieved curative response, but died some 2 years later of unknown cause (we did not have access to complete patient records); one achieved curative response, but eventually died due to debilitation and recurrent infections. Two had relapsed PCNSL, of whom one had reactions to MATRix treatment and was offered palliative WBRT, and one had disease progression despite MATRix treatment and was offered palliative WBRT.

We consecutively enrolled 65 healthy control subjects and assessed their eligibility for inclusion between 6/2018 and 3/2021. We excluded two healthy control subjects due to incidental brain MRI findings and one potential control subject due to suspected early‐stage Alzheimer's disease. Given the typical PCNSL onset amongst the elderly, we accepted elderly control participants who were taking antihypertensive medication. We thereby achieved age‐matching with 40 healthy control subjects.

### 2.1 Scanning, Image Reconstruction, and Physiological Monitoring

We performed fMRI using a Siemens Magnetom Skyra 3 T MRI scanner (Siemens Healthineers AG, Munich, Germany) with a 32‐channel head coil and an fMRI‐based magnetic resonance encephalography (MREG_BOLD_) sequence: TR = 100 ms, TE = 36 ms, FA = 25°, FOV = 192 mm, 5‐min scan, 3 mm isotropic voxel (Hennig et al. 2021; Assländer et al. 2013). Image reconstruction included a dynamic off‐resonance in k‐space (DORK) method, which corrected for scanner warming and respiration‐induced dynamic B_0_‐field changes (Hugger et al. 2011; Pfeuffer et al. 2002).

We recorded heart rate using the in‐scanner finger plethysmograph and respiration rate using the in‐scanner respiration belt. Importantly, the critically sampled 10 Hz temporal resolution of the MREG_BOLD_ sequence enables precise separation of the physiological signal components without aliasing (Kiviniemi et al. 2016; Huotari et al. 2019; Kananen et al. 2020; Tuovinen et al. 2020; Raitamaa et al. 2021; Rajna et al. 2021; Helakari et al. 2022; Järvelä et al. 2022; Poltojainen et al. 2025).

During the same session, we obtained high‐resolution T1‐weighted 3D images in line with the magnetization‐prepared rapid acquisition with gradient echo (MPRAGE) sequence without contrast‐enhancement: TR = 1900 ms, TE = 2.49 ms, TI = 900 ms, FA = 9°, FOV = 240 mm, 0.9 mm isotropic voxel. As per diagnostic routine, we also scanned the patients using a T2‐weighted fluid‐attenuated inversion recovery (FLAIR) sequence and a T1‐weighted gadolinium‐enhanced sequence to obtain a macroscopic estimation of tumor volumes. In seven patients, we obtained FLAIR datasets an average of 3 days before the MREG_BOLD_ acquisitions using standard imaging parameters on other scanners. Imaging parameters were as reported previously (Poltojainen et al. 2022).

### 2.2 Pre‐Processing

Pre‐processing was done according to standard FSL pipelines, identically to our previous studies (Poltojainen et al. 2022; Poltojainen et al. 2025). We used FLIRT to align the post‐processed MREG_BOLD_ maps to the standard 3 mm Montreal Neurological Institute (MNI 152) brain template. To exclude non‐brain voxels, we applied the resultant images to a binary brain mask including the ventricles. We performed further calculations using FSL 5.0.9, AFNI 20.1.2018, LIPSIA 3.1.0 (Lohmann et al. 2018), GraphPad Prism 9.1.0, and MATLAB software.

### 2.3 Extracting Physiological MREG_BOLD_ Frequency Bands for Further Pulsation Analysis

**FIGURE 1:**
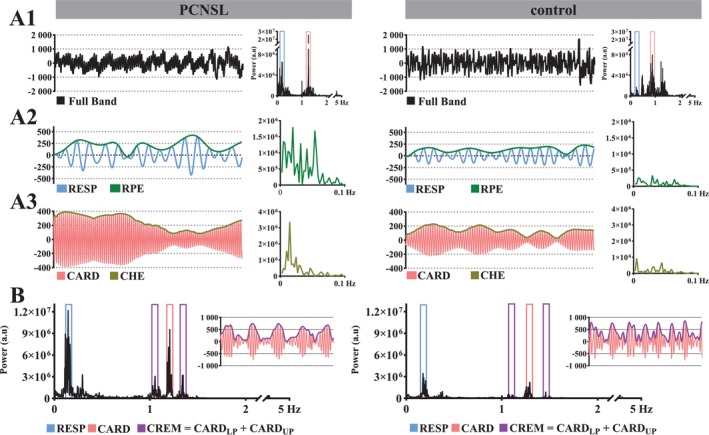
Method illustration. MREG_BOLD_ data from a representative primary central nervous system lymphoma (PCNSL) patient and from a typical healthy control subject. Sections A1–A3 represent data from a single voxel in the lumen of the anterior cerebral artery [*coordinates 0; 21; −9*]. (A1) The processed full band MREG_BOLD_ time‐series (*0.008–5 Hz; first 75 s of the 5‐min scan*) and respective full band frequency spectra. From these time‐series, the respiratory (RESP; *subject‐specific respiratory rate ± 0.05 Hz*) and cardiac (CARD; *subject‐specific cardiac rate ± 0.05 Hz*) time‐series are extracted for further analyses. (A2) An envelope is drawn over the peaks of the band‐passed RESP time‐series (*first 75 s of the 5‐min scan illustrated*) to create respiratory pulse envelope (RPE) waveforms at each voxel. The respective frequency spectrum (< 0.1 Hz) is shown on the right‐hand side. These RPE waveforms represent the low‐frequency variation in respiratory pulsation amplitude driven by the slow variations in the amplitude of each respiratory cycle. (A3) An envelope is drawn over the peaks of the band‐passed CARD time‐series (*first 75 s of the 5‐min scan illustrated*) to create cardiovascular pulse envelope (CHE) waveforms at each voxel. The respective frequency spectrum is shown (< 0.1 Hz) on the right‐hand side. These CHE waveforms represent the low‐frequency vasomotor activity causing variation in the cardiovascular pulsation amplitude within the brain. (B) Using the initial MREG_BOLD_ frequency spectra (0.008–5 Hz; whole‐brain spectra in this illustration), the lower *(CARD*_*LP*_ *) and upper (CARD*_*UP*_) heterodyne peaks are extracted from both sides of the principal cardiovascular power peak, and their amplitudes are summated to create voxel‐wise cardiorespiratory modulation (*CREM*) maps. As a “textbook example” of respiratory driven amplitude modulation of cardiovascular impulses, these heterodyne peaks are found at the subject‐specific cardiac rate ± subject‐specific respiratory rate. For illustration, the wide‐band cardiac MREG_BOLD_ time‐series (0.7–2 Hz) are shown with the respective CREM envelopes demonstrating how respiration is modulating the amplitude of cardiovascular brain impulses (*first 40 s of the 5‐min scan illustrated*).

As the (peri)arterial and (peri)venous pulsations are driven by cardiovascular (1 Hz) and respiratory (0.25 Hz) mechanisms respectively, their separation is obtainable simply by frequency band‐passing. Using the same methods as we have reported previously (Poltojainen et al. 2025), we extracted the voxel‐wise MREG_BOLD_ time‐series and the frequency spectra corresponding to respiratory (RESP; subject‐specific respiratory rate ± 0.05 Hz) and cardiovascular (CARD; subject‐specific cardiac rate ± 0.05 Hz) frequency bands for further assessment of the respective low‐frequency RPE and CHE pulsations (Figure 1).

Since we used finger photoplethysmography (PPG) frequency spectra, respiration belt frequency spectra, and global MREG_BOLD_ frequency spectra (0.008–5 Hz) to confirm cardiorespiratory frequency bands, we were able to verify subject‐specific cardiorespiratory rates with absolute precision. Technically, the cardiorespiratory frequency bands could have readily been determined from the unaliased MREG_BOLD_ data without need for external physiological measurements, as in (Poltojainen et al. 2025).

### 2.4 Extracting AF_RPE_ and AF_CHE_ to Assess Low‐Frequency BOLD Signal Fluctuations

We mapped RPE and CHE directly from the unaliased MREG_BOLD_ timeseries (Figure 1), as described previously (Tuunanen et al. 2024; Huotari et al. 2022). To this end, we used the MATLAB *findpeaks* function that finds the RESP and CARD signal maxima at each timepoint. Then, we used cubic interpolation to fit a curve to the extracted peaks. As a final step, we transformed the RPE and CHE waveforms to the frequency domain using the *3dPeriodogram* function at each voxel, followed by AF calculations from the spectra using the ALFF (amplitude of low‐frequency fluctuation) method shown previously (Raitamaa et al. 2021; Poltojainen et al. 2025; Raitamaa et al. 2024; Zang et al. 2007).

### 2.5 Calculating CREM Maps to Assess Respiratory Modulation Over Cardiac Fluctuations

We assessed CREM using the MREG_BOLD_ frequency spectra (Figure 1), as described previously (Raitamaa et al. 2021; Raitamaa et al. 2024). To form CREM maps, we first extracted the paired heterodyne peaks by applying the *fslroi* function on the initial MREG_BOLD_ frequency spectra (0.008–5 Hz); the heterodyne peaks arising from respiratory pulsations were positioned symmetrically about the subject‐specific cardiac rate. We set the width of both heterodyne peaks to 0.1 Hz, as the thermal noise outside the narrow peaks was minimal in visual inspection (Figure 1). Next, we calculated the respective amplitudes over the lower (AF_LP_) and upper (AF_UP_) heterodyne peaks and formed AF_CREM_ maps by summation of AF_LP_ and AF_UP_.

### 2.6 Formation of *Z*‐Score Maps

To examine physiological pulsations in individual subjects, we transformed their AF_RPE_, AF_CHE_, and AF_CREM_ maps to respective *Z*‐score maps, as described previously (Poltojainen et al. 2025). In brief, we subtracted the control population mean amplitude from the corresponding subject‐specific amplitude at each voxel and then normalized the difference with the control population standard deviation. Finally, we applied a *Z*‐score threshold of 3.0, such that the final *Z*‐score maps indicated voxels where the respective patient's amplitude value exceeded the age‐matched population control mean by at least three standard deviations.

To examine the group‐level properties of these *Z*‐score maps, we additionally formed cumulative incidence maps. To this end, we binarized the individual *Z*‐score maps at a threshold of 3.0, and then merged and summated the maps over the PCNSL group. In essence, these cumulative incidence maps depict the voxel‐wise number of PCNSL patients who demonstrated markedly increased MREG_BOLD_ amplitudes according to our *Z*‐scores ≥ 3.0 threshold. We formed a corresponding cumulative incidence map from the structural FLAIR imaging.

### 2.7 Assessment of Recurring Brain Areas With Increased MREG_BOLD_ Amplitudes

We were able to produce repeated MREG_BOLD_ scanning in a subset of PCNSL patients. Using this data, we assessed brain areas that recurrently showed increased MREG_BOLD_ amplitudes during treatment. For this, we used a variant of the above‐described cumulative incidence maps. First, we calculated the *Z*‐score maps by pulsation band from the repeat MREG_BOLD_ scans. We then binarized the individual maps at the threshold *Z*‐score ≥ 3.0 and merged the repeat scans of each patient for summation.

### 2.8 Calculating Pulsation Amplitudes Within Anatomical ROIs

Technically, due to the averaging process, classical voxel‐wise statistical analysis cannot entirely account for individual disease‐related alterations such as tumor locations, which can be variable. In this regard, region of interest (ROI) analysis may complement our voxel‐wise analyses. As such, we calculated the individual mean MREG_BOLD_ amplitude values (excluding any null voxel values) from the following ROIs: whole brain, lateral ventricles, grey matter (GM), and white matter (WM) using standard isotropic 3 mm MNI 152 templates. We defined the individual macroscopic tumor areas as oedematous hyperintensities upon FLAIR imaging, and segmented these volumes manually as described previously (Poltojainen et al. 2022). We next defined the adjacent peritumoral areas by dilating the binarized tumor masks using the *dilD* function. To enable placing our focus on parenchymal pulsations, we excluded the lateral ventricles from the peritumoral ROIs, and excluded macroscopic tumor ROIs from the GM and WM ROIs. Finally, we resampled all FLAIR‐images to isotropic 3 mm standard space to enable further calculations. We used the final ROI‐specific MREG_BOLD_ amplitudes in the subsequent survival analysis.

### 2.9 Clinical Factors in PCNSL

The Memorial Sloan Kettering Cancer Center (MSKCC) score has shown capability for PCNSL prognostics (Angelov et al. 2009; Abrey et al. 2006), whereby a higher MSKCC class predicts worse overall survival: Class 1 (age < 50 years), Class 2 (age ≥ 50 and Karnofsky performance score < 70), and Class 3 (age ≥ 50 years and Karnofsky performance score < 70). Other factors showing an association with worse overall survival in an albeit limited PCNSL series include a smaller number but greater volume of contrast‐enhancing tumor areas in gadolinium‐enhanced T1‐weighted MRI (Niparuck et al. 2019). We made a compilation of these clinical factors in our analysis.

### 2.10 Statistical Analysis

We examined the distributions of relevant parameters shown in Table 1 using normality testing within Prism and by visual inspection. We then compared differences in age, mean absolute head displacement, mean relative head displacement, framewise displacement, respiratory rate, and cardiac rate using exact two‐tailed Mann–Whitney U‐tests, also conducted in Prism, since these factors showed nonparametric distributions. We elected to consider effects of head displacement, since head motion may degrade data quality and interfere with the separation of physiological brain pulsations (Power et al. 2014). To test for differences in sex between the groups, we used a Fisher's two‐tailed exact test.

We examined voxel‐wise differences in MREG_BOLD_ amplitudes between the PCNSL and control subject groups using a nonparametric, threshold‐free permutation test (10,000 permutations) with false discovery rate (FDR) correction using the *vlisa_2ndlevel* function implemented in the LIPSIA software (Lohmann et al. 2018). We corrected these calculations for sex and mean absolute head motion, since only those parameters showed significant group differences.

Additionally, we compared the respective ROI‐specific MREG_BOLD_ amplitudes between the PCNSL patients and control subjects using exact two‐tailed Mann–Whitney U‐tests in Prism. As control subjects naturally lacked tumor areas, we compared the (peri)tumoral MREG_BOLD_ amplitudes of PCNSL patients against the respective whole‐brain amplitudes of control subjects and alternatively against the whole‐brain amplitudes of PCNSL patients.

To examine if the ROI‐specific MREG_BOLD_ amplitudes differentiated surviving PCNSL patients from PCNSL patients who had deceased by the end of follow‐up, we conducted separate receiver operating characteristics (ROC) area under the curve (AUC) analysis. Here we used the amplitudes that were able to differentiate deceased and surviving patients in further survival analysis, as described below. Additionally, we conducted an ROC analysis using the following clinical factors: MSKCC‐score (score between 1 and 3), age (years), *maximum* diameter of contrast‐enhancing foci (mm), *total* sum of all contrast‐enhancing tumor diameters (mm), number of contrast‐enhancing foci, and the volume of FLAIR hyperintensity (mm^3^).

To formally characterize the link between mortality and ROI‐specific MREG_BOLD_ amplitudes in PCNSL patients, we conducted multivariate Cox regression analysis in Prism. To this end, we coded the amplitudes as categorical values (below or above the group median) and included the following clinical factors within the mortality model: MSKCC score (score between 1 and 3), age (years), sex (female, male), *maximum* diameter of contrast‐enhancing foci (millimeters), and number of contrast‐enhancing foci. To examine the correlations between mean ROI‐specific MREG_BOLD_ amplitudes and clinical factors, we used nonparametric, paired Spearman coefficients. For all comparisons, we set the threshold for significance at *p* ≤ 0.05.

## 3 Results

### 3.1 Demographics

This retrospective case–control study included 30 pathologically diagnosed PCNSL patients and 40 healthy age‐matched control subjects. Diagnosis was confirmed by CSF aspiration in two (7%) patients, stereotactic needle biopsy in 17 (56%) patients, and excision in three (10%) patients. The exact method for biopsy (*stereotactic needle biopsy* versus *excision*) was not retrieved in eight (27%) cases, since these patients had been referred from other institutions after their confirmed diagnosis, and we did not have full access to their complete electronic patient records. In our previous work, we had visually noted that biopsy had minimal macroscopic effects in structural MRI (Poltojainen et al. 2022). In this population, seven PCNSL patients (23%) died during the study period. Patients and controls had some comorbidities (Table 1).

Through our rigorous selection process, we had obtained good age‐matching of control subjects [median = 62 years; interquartile range (IQR) = 61–66 years] with the PCNSL patient group (median = 66 years; IQR = 60–71 years) (*p* = 0.063), corresponding with the most common age of PCNSL diagnosis in Finland (Puhakka et al. 2022). The portion of females was higher amongst control subjects (73%) than amongst the PCNSL patients (30%) (*p* = 0.0006), partially reflecting greater PCNSL incidence in males (Puhakka et al. 2022). While the magnitude of mean *absolute* head displacement was significantly lower amongst control subjects (median = 0.161 mm; IQR = 0.113–0.219 mm) than amongst patients (median = 0.191 mm; IQR = 0.149–0.323 mm) (*p* = 0.022), the mean *relative* head displacement did not significantly differ between control subjects (median = 0.035 mm; IQR = 0.029–0.044 mm) and PCNSL patients (median = 0.036 mm; IQR = 0.032–0.044 mm) (*p* = 0.035). Notably, the magnitude of mean absolute and relative head displacement values remained low relative to the isotropic 3 mm voxel size. Similarly, there were no significant differences in framewise displacement between control subjects (median = 0.061 mm; IQR = 0.051–0.077 mm) and patients (median = 0.067 mm; IQR = 0.058–0.079 mm) (*p* = 0.21). There were no significant differences in respective respiratory rates between control subjects (median = 0.25 Hz; IQR = 0.21–0.29 Hz) and PCNSL patients (median = 0.25 Hz; IQR = 0.2–0.33 Hz) (*p* = 0.51). Similarly, there were no differences in cardiac rates between control subjects (median = 1.12 Hz; IQR = 1.03–1.25 Hz) and PCNSL patients (median = 1.19 Hz; IQR = 1.00–1.30 Hz) (*p* = 0.71). The groups had identical variability for mean subject‐specific respiratory rates (0.11–0.41 Hz), whereas there was more subject‐specific cardiac rate variability in control subjects (0.91–1.92 Hz) than in the PCNSL patients (0.78–1.38 Hz) (Table 1).

### 3.2 PCNSL Patients Have Brain‐Wide Increases in AF_RPE_

**FIGURE 2:**
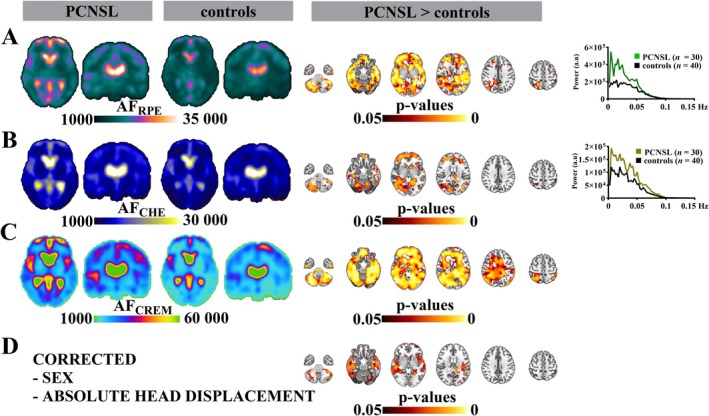
The amplitudes of respiratory pulse envelope (AF_RPE_), cardiovascular pulse envelope (AF_CHE_), and cardiorespiratory envelope modulation (AF_CREM_) are increased in primary central nervous system lymphoma (PCNSL) (*n* = 30) patients relative to healthy control subjects (*n* = 40). (A) Mean AF_RPE_ maps from these groups [*cursor in MNI coordinates: −9, −16, 4*], group average AF_RPE_ frequency spectra (0.008–0.15 Hz), and voxel‐by‐voxel statistical differences in AF_RPE_ according to a threshold‐free nonparametric test (*p ≤ 0.05; FDR‐corrected; no covariate correction*). (B) Mean AF_CHE_ maps from these groups, group average AF_CHE_ frequency spectra (0.008–0.15 Hz), and voxel‐by‐voxel statistical differences in AF_CHE_ according to a threshold‐free nonparametric test (*p ≤ 0.05; FDR‐corrected; no covariate correction*). (C) Mean AF_CREM_ maps from these groups and voxel‐by‐voxel statistical differences in AF_CREM_ according to a threshold‐free nonparametric test (*p ≤ 0.05; FDR‐corrected; no covariate correction*). (D) Only differences in AF_RPE_ persisted after correcting for the combined sex and absolute head displacement covariates (*p ≤ 0.05; FDR‐corrected*), despite these covariates showing no individual effects on any of the amplitude differences, as shown in Table S1. The anatomical background images are standard T1‐weighted Montreal Neurological Institute (MNI 152) templates.

In voxel‐wise brain mapping analysis, AF_CREM_, AF_CHE_, and AF_RPE_ were all increased in the PCNSL group (*p* ≤ 0.05; FDR‐corrected; no covariate correction) (Figure 2). In this analysis, AF_CREM_ showed the most spatially widespread increases in PCNSL, with AF_RPE_ and AF_CHE_ increases overlapping within watershed WM, cortical GM, and CSF areas. None of the pulsation mechanisms showed decreased amplitudes in PCNSL (data not shown).

Since there were differences in the sex distributions and absolute head displacement values between groups (Table 1), we assessed how these covariates individually affected the voxel‐wise amplitude differences between groups (Table S1). This comparison showed that after correcting for absolute displacement individually, all pulsation amplitudes remained increased, but after correcting for sex individually, only differences in AF_RPE_ and AF_CHE_ persisted between groups. After correcting for both covariates simultaneously, only the increases in AF_RPE_ persisted, while group differences in AF_CREM_ and AF_CHE_ diminished. Indeed, after correcting for both covariates simultaneously, the increases in AF_RPE_ were confined to the cerebellum, medulla oblongata, lateral ventricles, Sylvian fissures, watershed WM areas, and some bilateral deep brain regions (caudate nucleus, nucleus accumbens, corpus callosum, and hippocampus) (*p* ≤ 0.05; FDR‐corrected, corrected for sex and absolute head displacement) (Figure 2). These findings were mostly located in temporal brain regions. The increases in AF_RPE_ localized to the same areas that showed increased respiratory pulsation amplitudes in our previous study of these PCNSL patients (Poltojainen et al. 2025).

Considering that differences in AF_CREM_ diminished after correcting for sex, we assessed voxel‐by‐voxel covariate interactions with AF_RPE_, AF_CHE_, and AF_CREM_. In this analysis, the above‐mentioned covariates did not affect these amplitude parameters (data not shown).

### 3.3 ROI Analysis Shows That All Pulsation Amplitudes Are Increased in PCNSL

**FIGURE 3:**
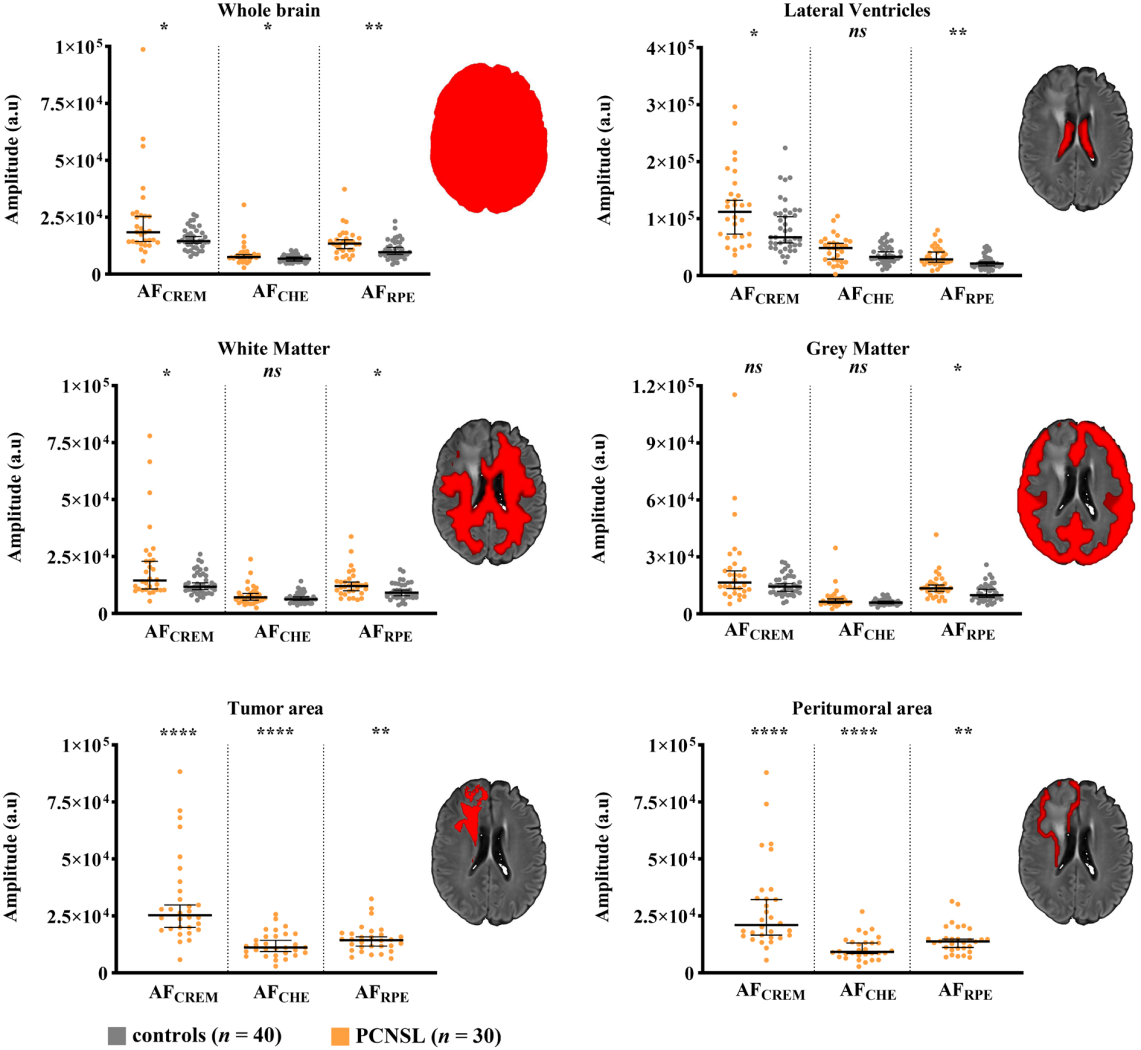
Region of interest (ROI) analysis shows that the amplitude of physiological fluctuations is widely increased in the brain of primary central nervous system lymphoma (PCNSL) patients. This figure illustrates average amplitudes over cardiorespiratory envelope modulation (AF_CREM_), cardiovascular pulse envelope (AF_CHE_), and respiratory pulse envelope (AF_RPE_) pulsation bands in PCNSL patients (*n* = 30) and in age‐matched control subjects (*n* = 40). Amplitudes are from multiple ROIs (here illustrated in one representative PCNSL patient): Whole brain, lateral ventricles, white matter, grey matter, patient‐specific tumor area, and patient‐specific peritumoral area. In PCNSL patients, the tumor areas were excluded from the white matter and grey matter analyses. Macroscopic tumor areas are defined as hyperintense FLAIR changes and peritumoral areas are defined as ring‐like areas surrounding those hyperintense FLAIR changes. Horizontal lines represent group median, and whiskers represent corresponding 95% Confidence Interval. The asterisk (*) denotes statistical significance between groups with respect to exact two‐tailed Mann Whitney U‐tests. Since control subjects lack comparative tumor areas and peritumoral areas, these PCNSL patients´ tumor‐related areas are compared against control subjects' whole‐brain amplitude values. For tabular results, please refer to Table S1.

According to our ROI analysis, AF_RPE_ was significantly higher in the PCNSL group than in the control group within the whole‐brain ROI, lateral ventricles, WM, and GM. AF_CREM_ was significantly increased within the whole‐brain ROI, WM, and lateral ventricles but not within GM. AF_CHE_ was significantly increased within the whole‐brain ROI but not within WM, GM, or lateral ventricles (Figure 3; Table S2).

Relative to the mean whole‐brain amplitudes in the *control* group, the (peri)tumoral ROIs in PCNSL patients had significantly increased AF_CREM_ and AF_CHE_ with lesser increases in AF_RPE_. Similarly, relative to the mean whole‐brain amplitudes in the *patient* group, the (peri)tumor ROIs in PCNSL patients demonstrated significant increases in AF_CHE_ and AF_CREM_ but not in AF_RPE_ (Table S2).

### 3.4 Tumor AF_RPE_ and Whole Brain AF_RPE_ Are Linked to Survival

**FIGURE 4:**
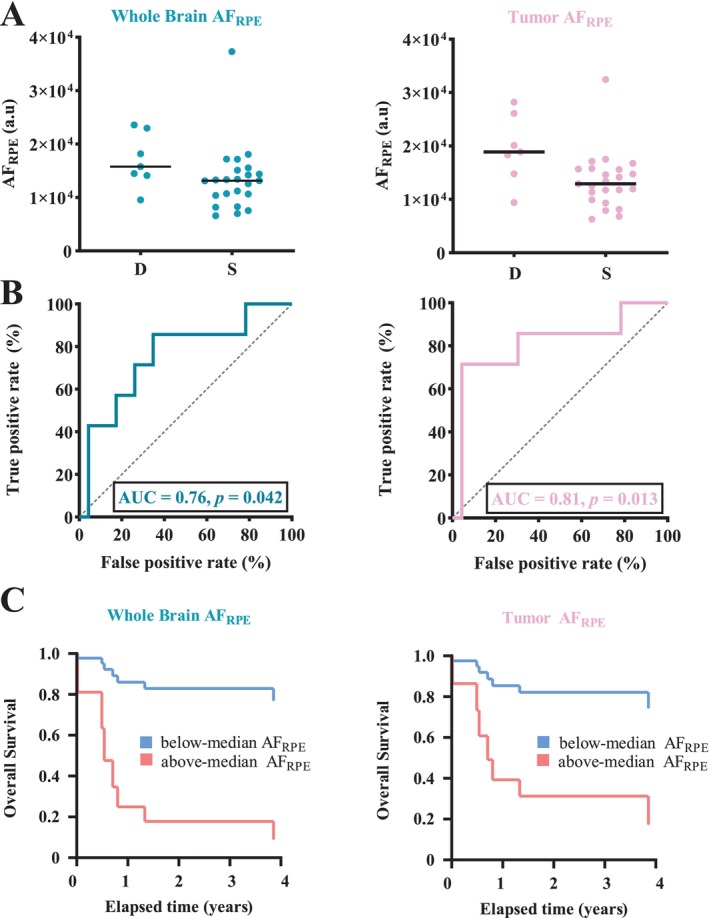
The amplitudes over the respiratory envelope modulation band (AF_RPE_) are linked to mortality in patients with primary central nervous system lymphoma (PCNSL). (A) Average region‐specific AF_RPE_ values in seven deceased (D) and 23 surviving (S) PCNSL patients. The horizontal lines represent group median values. (B) Receiver Operating Characteristics (ROC) area under curve (AUC) analysis shows that (only) these region‐specific AF_RPE_ differentiate subsequently deceased and surviving PCNSL patients. (C) A survival curve generated by a multivariate Cox regression model, with median AF_RPE_ value as a classifier.

We examined if ROI‐specific MREG_BOLD_ parameters could differentiate subsequently deceased and surviving PCNSL patients. In ROC analysis, we found that only whole‐brain AF_RPE_ (AUC = 0.76; 95% CI = 0.55–0.97; *p* = 0.042), WM AF_RPE_ (AUC = 0.76; 95% CI = 0.55–0.97; *p* = 0.037), tumor AF_RPE_ (AUC = 0.81; 95% CI = 0.60–1.00; *p* = 0.0133), and peritumoral AF_RPE_ (AUC = 0.79; 95% CI = 0.58–0.97; *p* = 0.023) could differentiate the seven deceased and 23 surviving PCNSL patients (Figure 4).

The following clinical factors did not differentiate deceased and surviving PCNSL patients: *maximum* diameter of contrast‐enhancing foci, *total* sum of all contrast‐enhancing tumor diameters, number of contrast‐enhancing foci, MSKCC score, age, sex, and volume for FLAIR hyperintensity.

Since whole‐brain AF_RPE_ and tumor AF_RPE_ were able to differentiate deceased and surviving patients, we assessed the link between these amplitudes and survival in further Cox regression analysis. In the first multivariate model, whole‐brain AF_RPE_ was linked to mortality based on the following hazard ratios (HRs): above‐median whole‐brain AF_RPE_ (HR = 9.19), increasing MSKCC score (HR = 3.90), increasing number of contrast‐enhancing foci (HR = 1.80), increasing *maximum* diameter of contrast‐enhancing foci (HR = 1.02), increasing age (HR = 0.90), female sex (HR = 0.40) (Figure 4). In the second multivariate analysis, tumor AF_RPE_ was also linked to mortality with the following HRs: above‐median tumor AF_RPE_ (HR = 5.91), increasing MSKCC score (HR = 2.96), increasing number of contrast‐enhancing foci (HR = 1.50), increasing *maximum* diameter of contrast‐enhancing foci (HR = 1.00), increasing age (HR = 0.93), female sex (HR = 0.33) (Figure 4).

### 3.5 AF_RPE_ and AF_CREM_ Correlate With the Number of Contrast‐Enhancing Foci

We determined the Spearman correlation coefficients (*r*_s_) between ROI‐specific MREG_BOLD_ amplitudes and clinical parameters (Table S3). AF_RPE_ from the lateral ventricles (*r*_s_ = 0.52, *p* = 0.003) and tumor areas (*r*_s_ = 0.39, *p* = 0.034) correlated with the number of contrast‐enhancing foci. Tumor AF_CHE_ correlated with maximum diameter of contrast‐enhancing area (*r*_s_ = −0.37, *p* = 0.045) and with volume for FLAIR hyperintensity (*r*_s_ = −0.40, *p* = 0.027), whereas WM AF_CHE_ correlated with age (*r*_s_ = 0.44, *p* = 0.014). AF_CREM_ from whole brain (*r*_s_ = 0.41, *p* = 0.026), lateral ventricles (*r*_s_ = 0.53, *p* = 0.003), tumor areas (*r*_s_ = 0.47, *p* = 0.009), and peritumoral areas (*r*_s_ = 0.38, *p* = 0.041) correlated with the number of contrast‐enhancing foci. WM AF_CREM_ (*r*_s_ = 0.43, *p* = 0.018) and peritumoral AF_CREM_ (*r*_s_ = 0.36, *p* = 0.048) correlated with age.

### 3.6 *Z*‐Score Mapping Reveals Increased Pulsation Amplitudes Within Tumor Areas, Brain Parenchyma, and CSF Spaces

Using the individual *Z*‐score maps as described above, we compared within individual PCNSL patients the areas of increased pulsation amplitude as opposed to control subjects (Figure S1). There was considerable overlap between zones of increased AF_RPE_, AF_CHE_, and AF_CREM_, especially in tumor areas. Increased AF_CREM_ and AF_RPE_ also extended beyond the macroscopic tumor margins to the adjacent WM and lateral ventricles. In contrast, PCNSL subjects did not have any amplitudes below 3.0 deviations (data not shown).

The group‐level cumulative incidence maps show that increased MREG_BOLD_ amplitudes extended beyond the macroscopic tumor areas, as assessed by hyperintensities in FLAIR imaging (Figure S2). Indeed, the macroscopically visible tumor areas were typically located within the periventricular WM, whereas increased MREG_BOLD_ amplitudes were generally located more distally to the ventricles. The *Z*‐score map of AF_CREM_ showed the most spatially widespread changes in high prevalence among the patients, with increased pulsation within periventricular WM, cortical GM, the thalami, and CSF areas. The prevalent areas of AF_CREM_ elevations were mostly configured in a temporo‐occipital distribution. The prevalence on increased AF_RPE_ was greatest within distal brain areas, but also within the bilateral caudate nuclei, while having a fronto‐temporal distribution that was comparable to the prevalence of increased cardiorespiratory pulsation in our previous study of these PCNSL patients (Poltojainen et al. 2025). The prevalence of increased AF_CHE_ was also greatest within distal areas, but also within the caudate nuclei, likewise having a frontal and occipital distribution that was comparable to the prevalence of increased cardiac pulsation seen in our previous study of these patients (Poltojainen et al. 2025).

### 3.7 Repeat Scans Show Some Fluctuation in MREG_BOLD_ Amplitude Values During Treatment

We assessed the variability of MREG_BOLD_ amplitudes over time by repeated scanning between treatment rounds when possible. These acquisitions yielded repeat scans in three deceased patients and 14 surviving patients. Overall, we obtained five repeat scans in one patient, four scans in two patients each, three scans in four patients each, and two scans in the remaining 10 patients. Considering all repeat scans, the median period between repeat scans was 28 days (IQR = 21–48 days), in line with the 3–4‐week intervals between treatment rounds.

The repeated MREG_BOLD_ data did not show any consistent trend of increasing or decreasing ROI‐specific pulsation amplitudes over the course of treatment; instead, the amplitudes remained relatively stable across time, albeit with some variability in individual subjects. Interestingly, deceased and surviving PCNSL patients showed comparable amplitude variations over time (Figure S3).

### 3.8 Increased MREG_BOLD_ Amplitudes Recur Within the Same Anatomical Areas in Repeated Scanning

Our summation maps in individual PCNSL patients revealed that increased AF_RPE_, AF_CHE_, and AF_CREM_ recurred mostly within tumor and CSF areas across repeated scanning (Figure S4). AF_CREM_ showed the most widespread changes in terms of recurringly increased pulsation amplitude, perhaps reflecting the fact that respiratory and cardiac signals invariably show strong pulsation in these areas, as described previously (Kiviniemi et al. 2016; Raitamaa et al. 2021; Poltojainen et al. 2025).

## 4 Discussion

In this study, we analyze AF_RPE_, AF_CHE_, and AF_CREM_ in PCNSL patients and in control subjects. Upon voxel‐wise analysis, PCNSL brains showed the most widespread increases in AF_CREM_, followed by AF_RPE_ and AF_CHE_. Increased AF_RPE_ was noted even after rigorous covariate correction. Moreover, whole‐brain AF_RPE_ and tumor AF_RPE_ were linked to mortality in PCNSL patients and correlated with the number of contrast‐enhancing foci. Increased AF_RPE_ was found within whole‐brain, WM, GM, CSF, and to a lesser degree in (peri)tumoral ROIs, while increased AF_CREM_ and AF_CHE_ were found most distinctly within (peri)tumoral ROIs, thus indicating differential contributions of respiratory‐driven venous and cardiovascular‐driven arterial brain pulsations in PCNSL. Upon assessment of individual PCNSL patients, *Z*‐score mapping showed similar findings of increased AF_RPE_, AF_CHE_, and AF_CREM_ overlapping with hyperintense FLAIR tumor changes, but with increased AF_CREM_ and AF_RPE_ extending beyond the macroscopic tumor margins to WM and lateral ventricles. Finally, *Z*‐score analysis of repeated MREG_BOLD_ scanning from 17 PCNSL patients showed persistently increased pulsation amplitudes within tumor and CSF areas, albeit with some degree of amplitude variation over time.

### 4.1 AF_RPE_ Reflects Increased Low‐Frequency Venous Pulsation in PCNSL

Historically, the low‐frequency T2*‐weighted BOLD fMRI signal has been utilized in mapping hemodynamic responses to either spontaneous or task‐evoked brain activations (Ogawa et al. 1992; Biswal et al. 1995; Bandettini et al. 1992). Increased BOLD signal is detected by susceptibility changes stemming from regional changes in deoxy/oxyhemoglobin ratio resulting from activation‐induced vasodilation and subsequent blood flow increase. Following neuronal activation, the release of K^+^ around juxtaneural arteries initiates a propagating hyperpolarization and subsequent rapid vasodilation towards upstream precapillary arterioles (Longden et al. 2017). A considerable component of the BOLD signal is thus attributed to local vasomotion followed by (peri)vascular fluid flow. These responses are regulated by the autonomic nervous system, local humoral and metabolic factors, and blood gas (*p*O_2_, *p*CO_2_) concentrations, which collectively comprise the mechanisms for autoregulation that maintains constant capillary blood flow (Whittaker et al. 2019; Willie et al. 2014; Katura et al. 2006; Sliwka et al. 2001; Obrig et al. 2000; Murphy et al. 2013; Akselrod et al. 1981; Berntson et al. 1997; Bolt et al. 2025). A recent bioluminescence microscopy study also revealed spontaneous, transiently occurring hypoxic pockets localizing near cortical veins (Beinlich et al. 2024). In another regulatory mechanism, transient drops in tissue *p*O_2_ increased red blood cell flow velocity by promoting cellular deformation (Wei et al. 2016). Any such fluctuations in tissue *p*O_2_ would likely manifest in fMRI BOLD signal changes.

Cerebral vasomotion is partly modulated by fluctuations in CBF and CBV that arise from respiration, more specifically from variations in *p*CO_2_ and breathing volume (Wise et al. 2004; Birn et al. 2006; Birn et al. 2008; Chang et al. 2009; Chang and Glover 2009; Verstynen and Deshpande 2011; Golestani et al. 2015; Golestani et al. 2016). Transient elevations in *p*CO_2_ trigger arterial vasodilation that increases CBF as measured with flow‐sensitive MRI (Kastrup et al. 1999; Halani et al. 2015) and positron emission tomography (Rostrup et al. 2000), in association with increased BOLD signal intensity (Kastrup et al. 1999; Halani et al. 2015; Rostrup et al. 2000; Sobczyk et al. 2014). While some authors have noted that the increase in CBF would be accompanied by diluted venous deoxyhemoglobin concentrations, leading to an increase in the classical susceptibility‐weighted BOLD signal (Wise et al. 2004; Chang and Glover 2009), others have suggested that the *p*CO_2_‐triggered increase in BOLD signal is mostly driven by flow changes, especially in fMRI sequences with low repetition times (Rostrup et al. 2000), as in the MREG_BOLD_ sequence used in this study. In either case, it is apparent that respiration modulates arterial CBV and CBF to a sufficient degree that low‐frequency respiratory‐related fluctuations can be noted in the fMRI signal, especially within arteries and GM (Wise et al. 2004; Birn et al. 2006; Golestani et al. 2015; Golestani et al. 2016; Kastrup et al. 1999; Salas et al. 2021). Others argued that CO_2_ fluctuations could not fully explain the respiratory‐driven vasomotor fluctuations and instead argued that the autonomic nervous system is responsible for a major component of these fluctuations, for example, by local interneurons or by noradrenergic projections arising from brainstem nuclei like the locus coeruleus (Bolt et al. 2025).

The increased CBV and CBF in response to elevation of *p*CO_2_ is characterized by CVR, which can be mapped using the total low‐frequency fMRI signal, among other imaging modalities (Bhogal et al. 2014; Liu et al. 2019). Additionally, the low‐frequency fMRI variations in respiration have been modelled by various approaches, including peak end‐tidal CO_2_ (PetCO_2_) graphs (Wise et al. 2004; Chang and Glover 2009; Golestani et al. 2015; Golestani et al. 2016), belt‐derived respiration volume per time (RVT) graphs (Shmueli et al. 2007; Birn et al. 2006; Birn et al. 2008; Chang and Glover 2009; Verstynen and Deshpande 2011; Golestani et al. 2015; Golestani et al. 2016; Kassinopoulos and Mitsis 2021), or directly from the fMRI data (Golestani et al. 2016; Salas et al. 2021). More recently, we extracted RPE waveforms directly from the respiratory MREG_BOLD_ data (Huotari et al. 2022). In the present study, we utilized the AF_RPE_ to model low‐frequency amplitude modulations in the respiratory brain pulsations that drive the (peri)venous fluid flow within the brain (Santisakultarm et al. 2012; Dreha‐Kulaczewski et al. 2015; Lloyd et al. 2020; Söderström et al. 2025). In effect, the AF_RPE_ reported in this study reflects how much the cerebral (peri)venous structures can pulsate due to changes in respiration, and the extent of modulation of venous blood volume and blood flow, and counteracting reciprocal oscillations within surrounding perivenous CSF spaces. Thus, the AF_RPE_ offers a physiological model of the CVR in the venous compartment. Alternatively, arterial CVR can be simultaneously modelled using the AF_CHE_ (Huotari et al. 2022).

In this study, voxel‐wise analysis showed increased AF_RPE_ extending across the brain of PCNSL patients. The areas of increased AF_RPE_ showed a significant degree of overlap with the areas showing increased vasomotor and respiratory pulsation in our previous study (Poltojainen et al. 2025). This is striking, as the measured respiratory rates themselves in PCNSL did not differ between PCNSL and control groups. Similarly, increased intracranial respiratory pulsations were reported in patients with epilepsy, despite unaltered cardiorespiratory rates (Kananen et al. 2020). Increased AF_RPE_ in PCNSL patients may be driven by an increase in respiratory‐driven (peri)venous CBF, CBV, and CSF oscillations. Whether increased AF_RPE_ in PCNSL reflects a compensatory mechanism for normalizing flow patterns or direct disease‐induced pathology remains to be established. In either event, we see evidence for an intracranial mechanism allowing increased low‐frequency respiratory fluctuations despite unaltered external breathing patterns.

Through our ROI‐analysis, we found PCNSL subjects having increased AF_RPE_ within WM, GM, and CSF areas that appeared structurally normal in clinical imaging, suggesting that the (peri)venous pulsation may be dysfunctional in these areas. While (peri)tumoral AF_RPE_ was modestly increased in (peri)tumoral areas of PCNSL subjects, the same areas showed more marked elevations in AF_CHE_ and AF_CREM_. At the same time, AF_CREM_ showed some elevations within CSF and WM whereas AF_CHE_ remained unaltered in other ROIs. These results suggest that the perivascular infiltration by lymphoma cells within the macroscopic tumor core evokes lesser increases in respiratory (peri)venous pulsations as compared to cardiovascular (peri)arterial pulsations. While this might reflect a difference in venous versus arterial densities, some other mechanism might be responsible for reducing respiratory fluctuations without concomitant reductions in cardiovascular fluctuations.

Our survival analysis showed that elevated tumor and whole‐brain AF_RPE_ were both linked to mortality in PCNSL patients, with both survival curves reaching a plateau after the first year of follow‐up. On the other hand, AF_CHE_ and AF_CREM_ were not linked to mortality. Despite our rigorous analysis, it remains unclear why exactly AF_RPE_ is related to mortality. For example, while tumor AF_RPE_ correlated with the number of contrast‐enhancing foci, AF_RPE_ did not correlate with macroscopic size estimates and did not systematically decrease in surviving PCNSL patients undergoing successful treatment. These findings could reflect that our AF_RPE_ does not directly reflect macroscopically tumor visible burden but rather an extent of (peri)venous dysfunction that may have a greater bearing on prognosis than arterial dysfunction.

Data from a subset of 17 PCNSL patients showed that AF_RPE_, AF_CHE_, and AF_CREM_ all remained elevated and relatively stable during treatment, albeit with some temporal variability in individual subjects. As the temporal variability in pulsation amplitudes did not markedly differ between deceased and surviving PCNSL patients, the pulsation amplitudes did not necessarily directly reflect macroscopically visible tumor burden but rather changes to vessel function. There may also be disease‐related and treatment‐related structural changes to the (peri)vascular environment despite successful eradication of lymphoma cells. In either case, the increased amplitudes recurred mostly in the same tumor and CSF areas that also showed increased respiratory and cardiovascular pulsation in our previous study. Our present findings support our initial hypothesis that AF_RPE_, AF_CHE_, and AF_CREM_ increases arise from physiological vasomotor, respiratory, and cardiovascular processes occurring within the CSF and blood vessels. Of note, these findings generally indicate stability of the MREG_BOLD_ technique across time.

In general, multiple factors may potentially contribute towards the increased AF_RPE_ in PCNSL patients. First, an associated deterioration of the BBB could have increased vessel wall compliance to interstitial fluid pulsation, whereas the occlusion and dilation of perivascular spaces could in contrast have diminished perivascular CSF flow. Consequently, altered (peri)venous pulsation could additionally manifest in altered CSF fluctuations (Wagshul et al. 2011). However, the extent of (peri)vascular changes in macroscopically normal‐appearing brain can be uncertain (Aho et al. 1993; Molnár et al. 1999). Second, pro‐inflammatory proteins such as cytokines could increase or decrease cerebrovascular reactivity in PCNSL patients (Sugita et al. 2015; O'Connor et al. 2019). Third, the regional loss or displacement of astrocytic end‐feet in PCNSL brain (Aho et al. 1993; Molnár et al. 1999) could impair the function of aquaporin 4 (AQP4) water channels that facilitate fluid flow, resulting in a net fluid stagnation within the brain, and consequently enlarged perivascular spaces as expressed by experimental data (Gomolka et al. 2023). Normally, the astrocytic AQP4 expression is higher around veins than arteries, perhaps leading to regionally differing susceptibility to impaired glymphatic clearance. In PCNSL, AQP4 expression is substantially elevated in macroscopic tumor regions and remains slightly elevated within normal‐appearing brain areas (Nico et al. 2012), perhaps reflecting a physiological response to normalize fluid flow. Regardless of the functional status of these water channels, increased AQP4 expression in PCNSL was associated with better prognosis (Takashima et al. 2023). It is known that PCNSL patients frequently present with neurocognitive and behavioral changes (Deutsch and Mendez 2015; Grommes et al. 2019), and poor performance status is an independent factor that is associated with poor prognosis in PCNSL (Angelov et al. 2009; Abrey et al. 2006). While in our present study the MREG_BOLD_ parameters were independent of performance status, dysfunctional autonomic nervous system might nonetheless contribute to these changes, as it has indeed been shown to modulate the vasomotor hemodynamic and CSF oscillations (Bolt et al. 2025).

Taken together, while we suppose that increased AF_RPE_ in PCNSL reflects impaired pulsation of the (peri)venous structures, the exact mechanisms by which intracerebral fluid flow is altered remain to be established.

### 4.2 AF_CHE_ Reflects Low‐Frequency Arterial Fluctuations in PCNSL

Physiologically, systemic cardiovascular factors are influenced by the autonomic nervous system, which controls blood pressure and peripheral vascular tone, heart rate, atrioventricular conduction, and cardiac contractility (Berntson et al. 1997). Arterial blood pressure is sensed within the aorta and carotid arteries by baroreceptors, and within the atria by mediators of the Bainbridge reflex, which together provide autonomic feedback modulation of cardiovascular function (Murphy et al. 2013; Berntson et al. 1997). Furthermore, the low‐frequency variations in respiratory and cardiac pulsations are intimately linked through autonomic pathways (Shmueli et al. 2007; Murphy et al. 2013; Chang and Glover 2009; Golestani et al. 2015). As such, low‐frequency variations in heart rate and blood pressure have been indicated as indirect markers for the balance between sympathetic and parasympathetic function, and for the neurovascular control of CBF and intracerebral pressure (Whittaker et al. 2019; Willie et al. 2014; Murphy et al. 2013; Berntson et al. 1997). Apart from these frequency variations, the intracerebral amplitude fluctuations in these cardiovascular waveforms are less well understood.

These systemic cardiovascular regulation factors have effects on brain physiology. Any fluctuations in blood pressure give rise to intracranial changes in CBV, CBF, and hemoglobin concentrations (Shmueli et al. 2007; Whittaker et al. 2019; Katura et al. 2006; Chang and Glover 2009; Kassinopoulos and Mitsis 2021; Tong et al. 2012). As such, peripheral PPG signals (Tong et al. 2012) and pneumatically measured arterial blood pressure fluctuations (Whittaker et al. 2019) tend to correlate with BOLD signal fluctuations although the peripheral recordings are not perfectly representative of central signal. While CBF is inextricably related to the systemic cardiovascular factors, it is also subject to local regulation by vasomotion of the smooth muscle cells embedded within the arterial wall (Whittaker et al. 2019; Willie et al. 2014; Katura et al. 2006; Sliwka et al. 2001; Obrig et al. 2000). For instance, a drop in arterial blood pressure is met with rapid increases in the global BOLD signal amplitudes in rat brain, whereas blood volume replenishment largely normalizes this BOLD signal increase (Kannurpatti et al. 2008). In fact, the amplitude of overall vasomotor fluctuations generally increases during various challenges that compromise brain homeostasis (Julien 2006). Collectively, these findings support an autoregulatory mechanism maintaining constant CBV and CBF, irrespective of respiration or CO_2_‐induced vasodilation. For example, visual stimulation without respiratory alterations in mice increased the regional vasomotion of brain vasculature, while also increasing the venous drainage of blood (van Veluw et al. 2020). Previous studies in human subjects likewise found increased BOLD signal and increased CBF in response to visual stimulus (Kastrup et al. 1999; Rostrup et al. 2000). Similarly, our group recently showed that visual cortex activation initially led to increased AF_CHE_, followed by increased overall BOLD signal in the visual cortex after some 1.3 s, whereas AF_RPE_ was unaffected by the stimulus (Huotari et al. 2022). Notably, in that study, the duration of BOLD response widened after activation, consistent with the notion proposed elsewhere that CBF and BOLD responses should lengthen during vasodilation (Halani et al. 2015). These results support the hypothesis that AF_CHE_ reflects arterial vasomotion and resultant increases in CBV and CBF. Additionally, the vessel contraction during vasomotion would transiently elevate the regional blood pressure, bringing faster conduction of cardiovascular impulses (Myllylä et al. 2011).

Be that as it may, systemic and local cardiovascular fluctuations seem to give rise to fluctuations in CBV, CBF, and hemoglobin concentrations, which in turn reflect upon the low‐frequency fMRI signal. The low‐frequency variations in the amplitude of cardiovascular fluctuations have traditionally been extracted from finger plethysmograph data, that is, from peripheral signal (Shmueli et al. 2007; Katura et al. 2006; Chang et al. 2009; Verstynen and Deshpande 2011; Golestani et al. 2016; Kassinopoulos and Mitsis 2021). More recently, we have extracted the low‐frequency cardiovascular vasomotor waveforms directly from the cardiovascular MREG_BOLD_ data, that is, from central signal (Huotari et al. 2022). We now utilize the AF_CHE_ to model low‐frequency amplitude modulations in the cardiovascular‐driven brain fluctuations that drive brain‐wide (peri)arterial fluid flow (Mitchell et al. 2011; Posse et al. 2013; Dagli et al. 1999; Bhattacharyya and Lowe 2004). In effect, our AF_CHE_ signal reflects the extent of pulsation of cerebral (peri)arterial structures, and the modulation of arterial blood flow and blood volume in response to altered cardiovascular fluctuation and counteracting reciprocal oscillations within surrounding periarterial CSF spaces. Thus, our AF_CHE_ metric offers a physiological model of the CVR on the arterial compartment.

Our present voxel‐wise analysis showed increased AF_CHE_ co‐localizing with some areas that had increased vasomotor and cardiovascular pulsation in our previous study (Poltojainen et al. 2025). There were no differences in heart rates between control and patient groups. While AF_CHE_ remained significantly increased in the PCNSL patients after separately adjusting for sex and absolute head displacement, joint adjustment for both covariates rather diminished the group differences in voxel‐wise AF_CHE_. However, our ROI‐analysis showed markedly increased AF_CHE_ within macroscopically visible (peri)tumoral ROIs, which showed lesser increases in AF_RPE_. In contrast, the ROI‐analysis did not show increased AF_CHE_ within other parenchymal regions that, in contrast, had markedly increased AF_RPE_. Overall, present results indicate an increased amplitude of low‐frequency cardiovascular pulsations in the cerebral arteries of PCNSL patients. Increased AF_CHE_ was most apparent within macroscopic (peri)tumoral areas and CSF areas, whereas there was a more global increase in AF_RPE_. This discrepancy suggests that the amplitudes of intracranial cardiovascular pulses are modulated differently than the respiratory pulses.

Perfusion imaging can reveal PCNSL tumor surface areas having CBV values much like those of normal GM (Xing et al. 2014; Abreu et al. 2024; Liao et al. 2009; Mansour et al. 2014). However, while surface areas of macroscopic PCNSL tumors may have high perfusion, this does not hold for the tumor core, as classical angiography studies show a diffuse, cloud‐like blush with some penetrating arteries on the surface of PCNSL tumors with lesser core perfusion (Jack et al. 1986). As such, PCNSL lesions organize in angiocentric cuffs, and find alternate vascular supplies (Ribatti et al. 2023).

We suppose that higher AF_CHE_ and AF_CREM_ in the tumor core may reflect reduced core vascularization, altered perfusion, and related increases in vasomotion. Since arteries are typically more capable of vasomotion than veins, this could perhaps partly explain why AF_CHE_ is more elevated within the macroscopic tumor cores than AF_RPE_. However, we have no information about the relative vessel densities of arteries and veins in PCNSL. Alternatively, since there is marked oedema, BBB disruption, and perivascular infiltration within the tumor core, elevated AF_CHE_ might reflect disturbed flow control through exaggerated variations in (peri)arterial resistance to fluid influx that is normally driven by cardiovascular pulsations. Such an increased resistance to periarterial flow changes could explain our finding of increased AF_CHE_ within CSF as well. Thirdly, the dense tumor areas may also support more rapid conduction of cardiovascular pulsations, resulting in increased regional flow speeds and the luminal narrowing due to vasomotion or perivascular PCNSL infiltration would transiently elevate the regional blood pressure, bringing faster conduction of cardiovascular impulses (Myllylä et al. 2011).

### 4.3 AF_CREM_ May Reflect Intracranial CSF and Blood Flow Fluctuations

The typically 0.25 Hz respiration modulates beat‐to‐beat variations in heart rate through a mechanism known as respiratory sinus arrhythmia (RSA) (Berntson et al. 1997). Physiologically, RSA actively modulates cardiac output and may improve pulmonary gas exchange and reduce energy expenditure by matching perfusion and ventilation (Yasuma and Hayano 2004). Previously, phase‐contrast MRI and echo planar fMRI have been used to characterize how respiration modulates the systolic flow speed of CSF and blood in the brain (Lloyd et al. 2020; Klose et al. 2000; Friese et al. 2004; Brooks et al. 2008). The more recently described amplitude modulation (AF_CREM_) is obtained with fast fMRI measurements (Raitamaa et al. 2021; Brooks et al. 2008). AF_CREM_ seems to originate from intracranial respiratory‐driven pressure changes, and the resulting modulatory AF_CREM_ wave has a spatiotemporal pattern that is distinct from those of cardiac and respiratory pulsations. The AF_CREM_ wave starts from the posterior fossa along the trajectory of inflowing CSF. Indeed, the AF_CREM_ and cardiac fluctuations dominate within major vessels and venous sinuses, central and spinal CSF spaces (Raitamaa et al. 2021; Brooks et al. 2008) whereas vasomotor fluctuations dominate within cortical GM and respiratory fluctuations dominate within periventricular WM (Raitamaa et al. 2021).

The physiological function of AF_CREM_ within the intracranial and spinal space is not yet fully understood. AF_CREM_ seems to reflect a form of interference whereby inhalation rhythmically increases, and expiration rhythmically decreases the cardiovascular brain impulses within both CSF and brain tissue. The inflow of arterial blood during cardiac contractions is counterbalanced by outflow of venous blood during inspiration, thus maintaining equilibrium in cerebral pressure within the rigid bony cranium, as described by the Monro‐Kellie doctrine. As such, AF_CREM_ may act to counterbalance momentary intracranial pressure and blood pressure fluctuations by linking venous return, cardiovascular arterial tone, and cardiac output (Lloyd et al. 2020; Söderström et al. 2025; Brooks et al. 2008).

Our present voxel‐wise analysis showed AF_CREM_ having the most spatially widespread increases in PCNSL patients. While the difference in AF_CREM_ between groups was robust to absolute head displacement, separate adjustment of sex diminished the group differences in AF_CREM_. ROI analysis revealed that AF_CREM_ was increased everywhere except in GM. The AF_CREM_, like AF_CHE_, was especially increased within the macroscopically visible (peri)tumoral areas. The subject‐wise *Z*‐score analysis similarly showed increased AF_CREM_ within macroscopic tumor core areas, with increases extending beyond the margins of those tumor areas to periventricular WM, cortical GM, and CSF areas. The group‐level *Z*‐score analysis showed the highest prevalence of increased AF_CREM_ within periventricular WM, cortical GM, the thalami, and CSF areas. Taken together, we suppose that the increased AF_CREM_ reflects altered interactions between respiratory and cardiovascular fluctuations, especially within (peri)tumoral and CSF areas.

### 4.4 Strengths, Limitations, and Prospects

Among the limitations of this study, our PCNSL patients and controls had some comorbidities (Table 1). However, group differences were robust to possible effects of age, relative head displacement, framewise head displacement, respiratory and cardiac frequency. Additionally, we used sex and absolute head displacement as covariates since these parameters showed differences between groups. Importantly, the stereotactic needle biopsy had minimal structural or anatomical effects on MRI in this population, as we had previously reported (Poltojainen et al. 2022). The retrospective nature of this study may call for some caution in the interpretation of the apparent link between AF_RPE_ and patient mortality. A prospective setting with a larger sample size could better validate the MREG_BOLD_ procedure as a prognostic tool in PCNSL. There was an abundance of female participants in the control group, and after correcting for sex, only AF_RPE_ and AF_CHE_ remained, which would suggest that sex has an effect on how intracerebral cardiovascular pulsations are modulated. However, further voxel‐by‐voxel assessment of covariate interactions revealed that sex did not markedly affect AF_CREM_.

As stated above, the AF_RPE_, AF_CHE_, and AF_CREM_ that are acquired using high‐frequency fMRI are likely influenced by flow‐related factors and classical, susceptibility‐related BOLD signal variations. In general, the BOLD signal is sensitive to nonneural sources including variations in CBV, CBF, blood gas concentrations, and hemoglobin levels. In future studies, one might attempt to increase the weighting of flow‐related BOLD signal effects using multi‐echo acquisition schemes (Whittaker et al. 2019; Glover et al. 1996). Additionally, nonstationary blood and CSF flow dynamics within the brain could be investigated by exploiting alternate fMRI analysis methods such as recursive regression (Tong and Frederick 2014), quasi‐periodic analysis (Kiviniemi et al. 2016; Raitamaa et al. 2021), or vector‐based analysis (Rajna et al. 2021).

As stated above, the high‐frequency MREG_BOLD_ acquisition enables precise measurement of brain pulsations without need for external recordings (Kiviniemi et al. 2016; Huotari et al. 2019; Kananen et al. 2020; Tuovinen et al. 2020; Raitamaa et al. 2021; Rajna et al. 2021; Helakari et al. 2022; Järvelä et al. 2022; Tuunanen et al. 2024; Poltojainen et al. 2025). Externally recorded PetCO_2_, RVT, PPG, ECG, and blood pressure waveforms would each reflect unique physiological properties while sometimes sharing physiological mechanisms. For instance, while both RVT and PetCO_2_ recordings may reflect shared mechanisms of intravascular CO_2_ fluctuations, RVT recordings reflect more directly the chest volume changes and are therefore suited to model unique physiological and magnetohydrodynamic properties extending beyond CO_2_ levels (Chang and Glover 2009; Golestani et al. 2015). The PPG signal used for heart rate monitoring also reflects blood volume and pressure effects (Kassinopoulos and Mitsis 2021), and while heart rate and blood pressure both modulate hematocrit, the effects of heart rate seem stronger (Katura et al. 2006). While we suggest that AF_CHE_ reflects regional vasomotion, the extent of systemic effects on AF_CHE_ remains to be further elucidated. As such, it remains a task for future studies to assess the differential effects of systemic cardiorespiratory factors on intracranial AF_RPE_ and AF_CHE_ using multimodal physiological recordings in conjunction with unaliased fMRI acquisition. Also, possible covariations between low‐ and high‐frequency fluctuations remain to be investigated as variations in respiratory and cardiac pulsations are intimately linked (Shmueli et al. 2007; Murphy et al. 2013; Chang and Glover 2009; Golestani et al. 2015). Finally, we note that while the modulation of vasomotor fluctuations by dysfunctional autonomic nervous system is likely in PCNSL, it cannot be further elucidated in this study but would be of interest in the future.

## 5 Conclusion

We found widespread increases in AF_CREM_ and AF_RPE_ across the brain of PCNSL patients, and lesser increases in AF_CHE_. Increased AF_RPE_ was robust to rigorous covariate correction and was linked to mortality. Increased AF_RPE_ was more notable within WM, GM, and CSF, with lesser increases within (peri)tumoral regions, whereas AF_CHE_ and AF_CREM_ increases were more pronounced within the macroscopic (peri)tumoral areas. Our AF_RPE_ metric may reflect pulsation of the (peri)venous compartment in response to respiratory pulsations, whereas AF_CHE_ more reflects the pulsation of the (peri)arterial compartment in response to cardiovascular pulsations. We speculate that the AF_CREM_ signal originates from intracranial respiratory‐driven pressure changes. Our findings highlight the potential of neurophysiological monitoring in CNS malignancies extending beyond traditional structural assessments, which could serve as early functional biomarkers for disease presence or progression, potentially preceding structural changes visible on conventional imaging. Moreover, as these pulsations are drivers of glymphatic fluid convection within the brain, understanding these disruptions in PCNSL could potentially inform optimization of the delivery of antineoplastic drugs.

## Author Contributions

Conception of study: Valter Poltojainen, Matti Järvelä, Janne Kananen, Heta Helakari, Vesa Kiviniemi. Data acquisition: Valter Poltojainen, Nina Keinänen, Juha‐Matti Isokangas, Hanne Kuitunen, Juha Nikkinen, Vesa Korhonen, Tommi Kalevi Korhonen, Sami Tetri, Outi Kuittinen, Vesa Kiviniemi. Analysis: Valter Poltojainen, Vesa Korhonen, Niko Huotari, Lauri Raitamaa. Interpretation: Valter Poltojainen, Michaela K. Bode, Heta Helakari, Vesa Kiviniemi. All authors have contributed to the draft of the manuscript and have approved its final version as submitted. The authors have agreed to be accountable for all aspects of this work and are committed to addressing properly any questions that might arise in relation to the accuracy or integrity of this work.

## Funding

This work was supported by the Academy of Finland (TERVA funding 314497; TERVA funding 335720; Project 338599 funding), the Arvo and Lea Ylppö Foundation (MJ), the Emil Aaltosen Säätiö (MJ; LR), the Finnish Brain Foundation (LR, JKa), the Finnish Cultural Foundation (TKKo), the Finnish Medical Foundation (VP, JKa, TKKo), the Finnish Medical Society Duodecim (MJ), the Instrumentarium Science Foundation (MJ, JKa), the Jane and Aatos Erkko Foundation (2016‐2020 #210043), the Juhani Aho Foundation for Medical Research (LR), Maire Taposen Säätiö (LR, JKa), the Medical Research Center Oulu (JKa), the Orion Research Foundation (VP, MJ, TKKo), the Tauno Tönningin Säätiö (LR).

## Ethics Statement

This study adheres to the Declaration of Helsinki and institutional approval was granted by the Ethical Committee of Northern Ostrobothnia Hospital District, Oulu University Hospital.

## Consent

Written informed consent was obtained from all subjects.

## Conflicts of Interest

The authors declare no conflicts of interest.

## Supporting information


**Data S1:** Supporting information.

## Data Availability

The data that support the findings of this study are available from the corresponding author upon reasonable request.
