# Supplementary material for: The Regulation of Vasomotor and Cardiorespiratory Pulsations Is Disrupted in Primary Central Nervous System Lymphoma: A Case–Control fMRI Study
Source: Hum Brain Mapp. 2026 Mar 11;47(4):e70495. doi: 10.1002/hbm.70495 (PMC13093760; doi:10.1002/hbm.70495)
Supplement: Supplementary file 1 — Data S1: Supporting information. [file HBM-47-e70495-s001.docx]

**The regulation of vasomotor and cardiorespiratory pulsations is disrupted in primary central nervous system lymphoma: A case-control fMRI study**

Valter Poltojainen MD^1,2,3^, Matti Järvelä MD^1,2,3^, Nina Keinänen MD^4^, Michaela K. Bode MD, PhD^5^, Juha-Matti Isokangas MD, PhD^5^, Hanne Kuitunen MD, PhD^6^, Juha Nikkinen MSc, PhD^5,7^, Vesa Korhonen MSc, PhD^1,2,3^, Niko Huotari MSc^1,2,3^, Lauri Raitamaa MSc^1,2,3^, Janne Kananen MD, MSc, PhD^1,2,3,8^, Heta Helakari MSc, PhD^1,2,3^, Tommi Kalevi Korhonen MD, PhD^9^, Sami Tetri MD, PhD^9^, Outi Kuittinen MD, PhD^10,11^, Vesa Kiviniemi MD, PhD^1,2,3,5^

**Affiliations**

1. Oulu Functional NeuroImaging-(OFNI), Research Unit of Health Sciences and Technology, University of Oulu, Oulu, Finland
2. Medical Imaging, Physics and Technology (MIPT), Faculty of Medicine, University of Oulu, Oulu, Finland
3. Medical Research Center (MRC), Oulu University Hospital, Oulu, Finland
4. Anesthesiology, Oulu University Hospital, Oulu, Finland
5. Department of Diagnostic Radiology, Oulu University Hospital, Research Unit of Health Sciences and Technology (HST), University of Oulu, Oulu, Finland
6. Oncology and Hematology, Oulu University Hospital, Oulu, Finland
7. Oncology and Radiotherapy, Oulu University Hospital, Oulu, Finland
8. Clinical Neurophysiology, Oulu University Hospital, Oulu, Finland
9. Neurosurgery, Clinical Neuroscience, University of Oulu/Oulu University Hospital
10. Cancer Center, Kuopio University Hospital, Kuopio, Finland
11. Faculty of Health Medicine, Institute of Clinical Medicine, University of Eastern Finland, Kuopio, Finland

**Corresponding author**

Valter Poltojainen

E-mail: Valter-Ari.Poltojainen@oulu.fi

**Supplementary Figures**

**
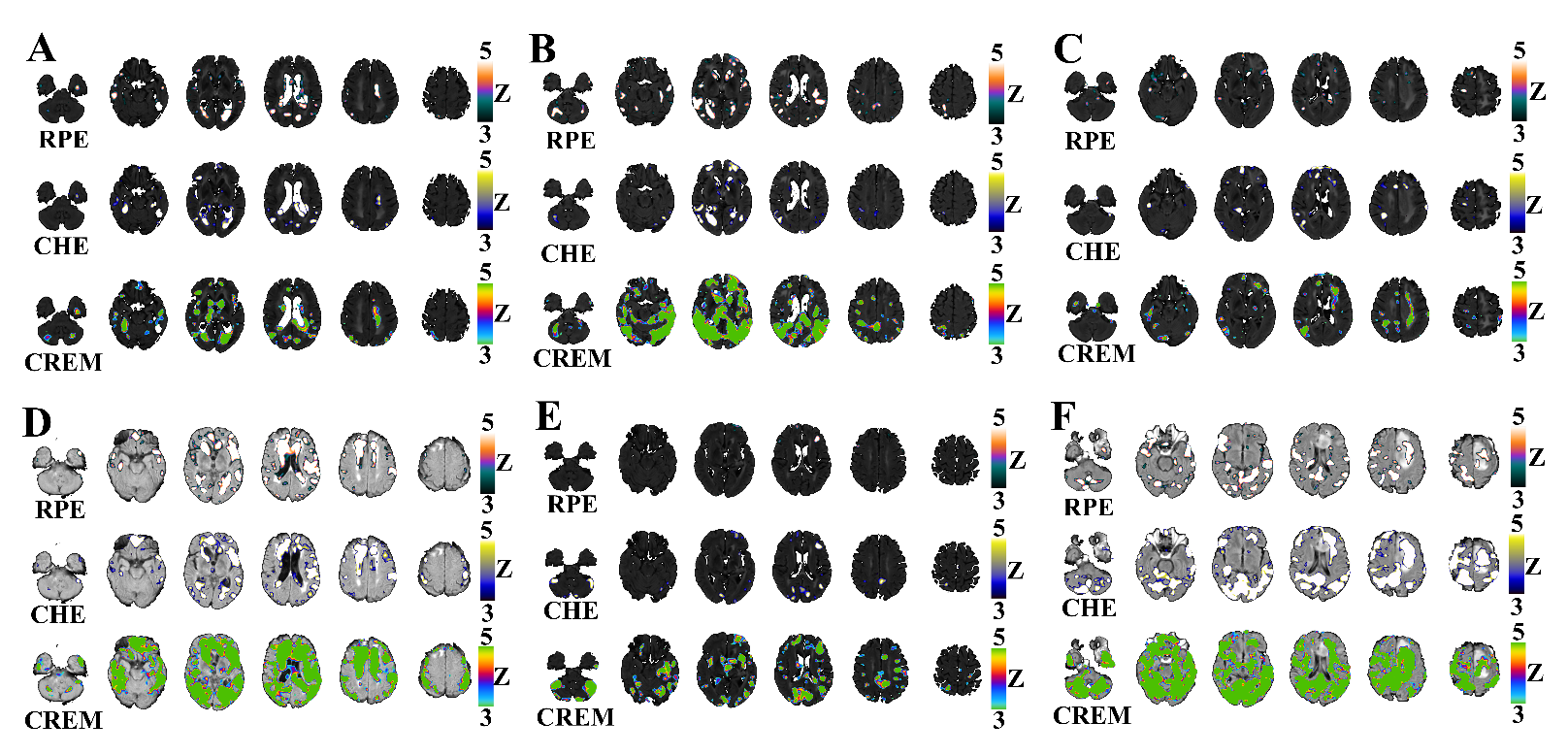
**

**Supplementary Figure 1. Subject-level mapping of MREG_BOLD_ amplitude changes shows increased pulsation all over the brain of primary central nervous system lymphoma** (**PCNSL) patients.** This figure illustrates Z-score-encoded respiratory pulse envelope (AF_RPE_), cardiovascular pulse envelope (AF_CHE_), and cardiorespiratory envelope (AF_CREM_) maps in six PCNSL patients (panels A to F). Colored voxels represent areas where the corresponding metric value exceeds, in that voxel, the control population mean by at least three standard deviations (Z ≥ 3). The anatomical background images are patient-specific T2-weighted FLAIR images that demonstrate edematous tumor areas.

**
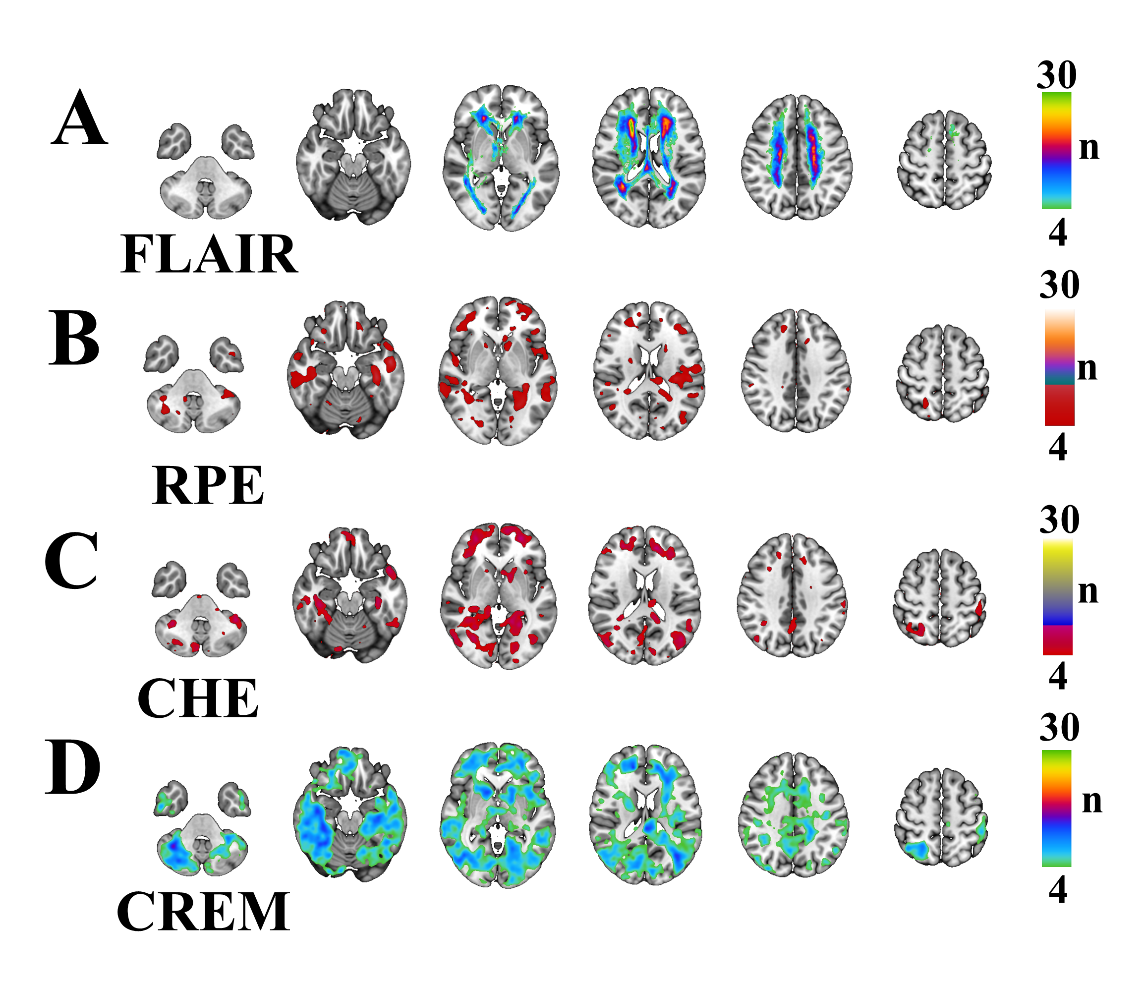
**

**Supplementary Figure 2. The cumulative incidence of MREG_BOLD_ findings and FLAIR findings in primary central nervous system lymphoma (PCNSL) patients (*n* = 30). (A)** The number of PCNSL patients (4 to 30) demonstrating macroscopic tumor oedema at a given voxel, as assessed by T2-weighted fluid-attenuated inversion recovery (FLAIR) imaging. **(B)** The number of PCNSL patients (4 to 30) demonstrating amplitude of respiratory envelope modulation (AF_RPE_) Z-scores exceeding 3.0 at a given voxel. This cumulative incidence map is formed by first thresholding the AF_RPE_-derived Z-score map at 3.0 and binarizing the voxel values. The binary maps are merged across the group followed by summation. **(C)** The number of PCNSL patients (4 to 30) demonstrating amplitude of cardiovascular pulse envelope modulation (AF_CHE_) Z-scores exceeding 3.0 at a given voxel. **(D)** The number of PCNSL patients (4 to 30) demonstrating amplitude cardiorespiratory envelope modulation (AF_CREM_) Z-scores exceeding 3.0 at a given voxel. The background images are standard T1-weighted Montreal Neurological Institute templates.

**
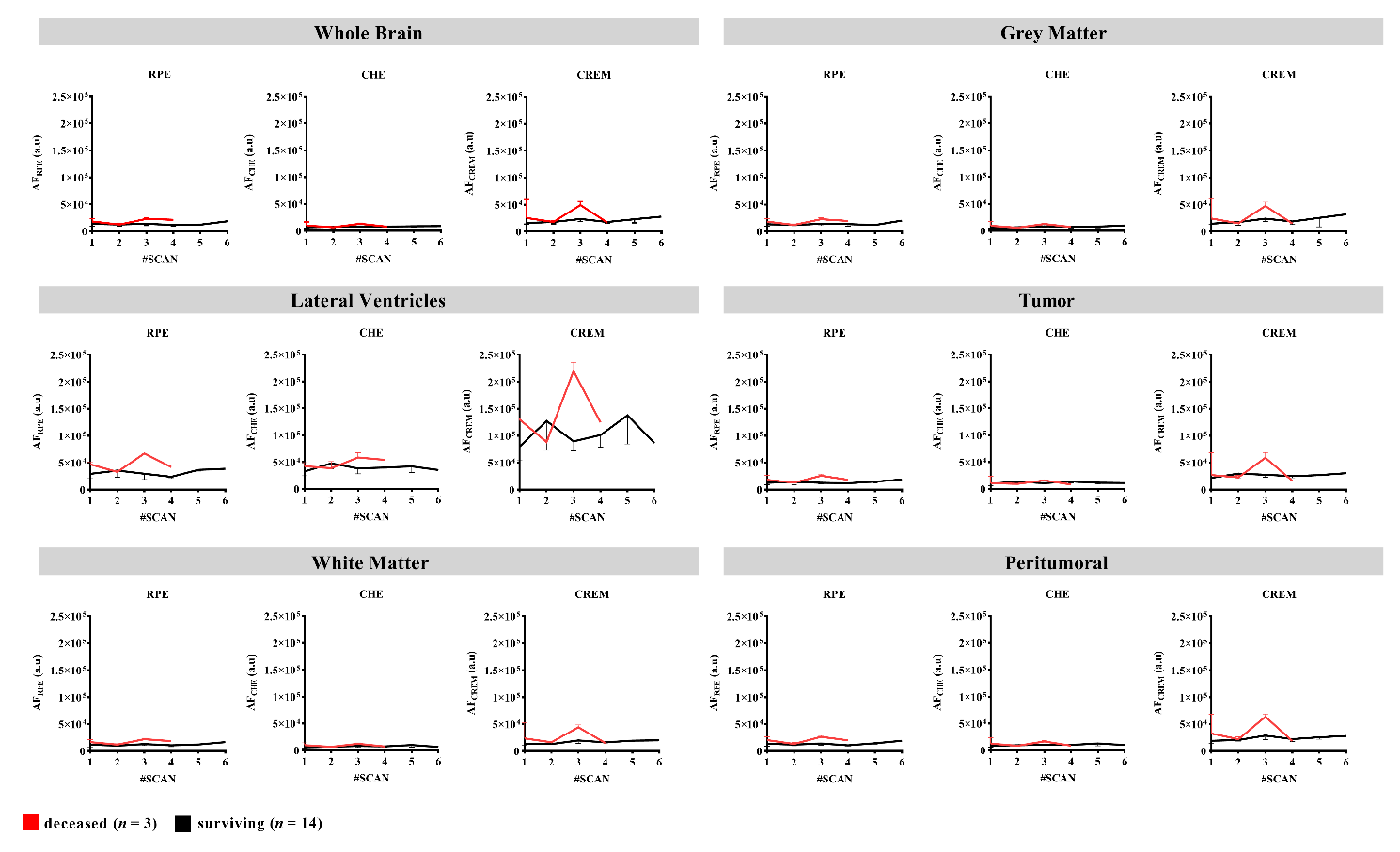
**

**Supplementary Figure 3. MREG_BOLD_ amplitudes from repeat scanning in deceased (*n* = 3) and in surviving (*n* = 14) primary central nervous system lymphoma (PCNSL) patients.** Each panel illustrates *mean* amplitude values from the following regions of interest: whole brain, lateral ventricles, white matter, grey matter, macroscopic tumor areas as assessed by hyperintensity in FLAIR imaging, and peritumoral regions. The connecting lines represent median whole-brain amplitude values at each time point and the whiskers represent corresponding interquartile ranges. CREM (*cardiorespiratory envelope modulation*), CHE (*cardiovascular pulse* *envelope*), RPE (*respiratory pulse envelope*).

**
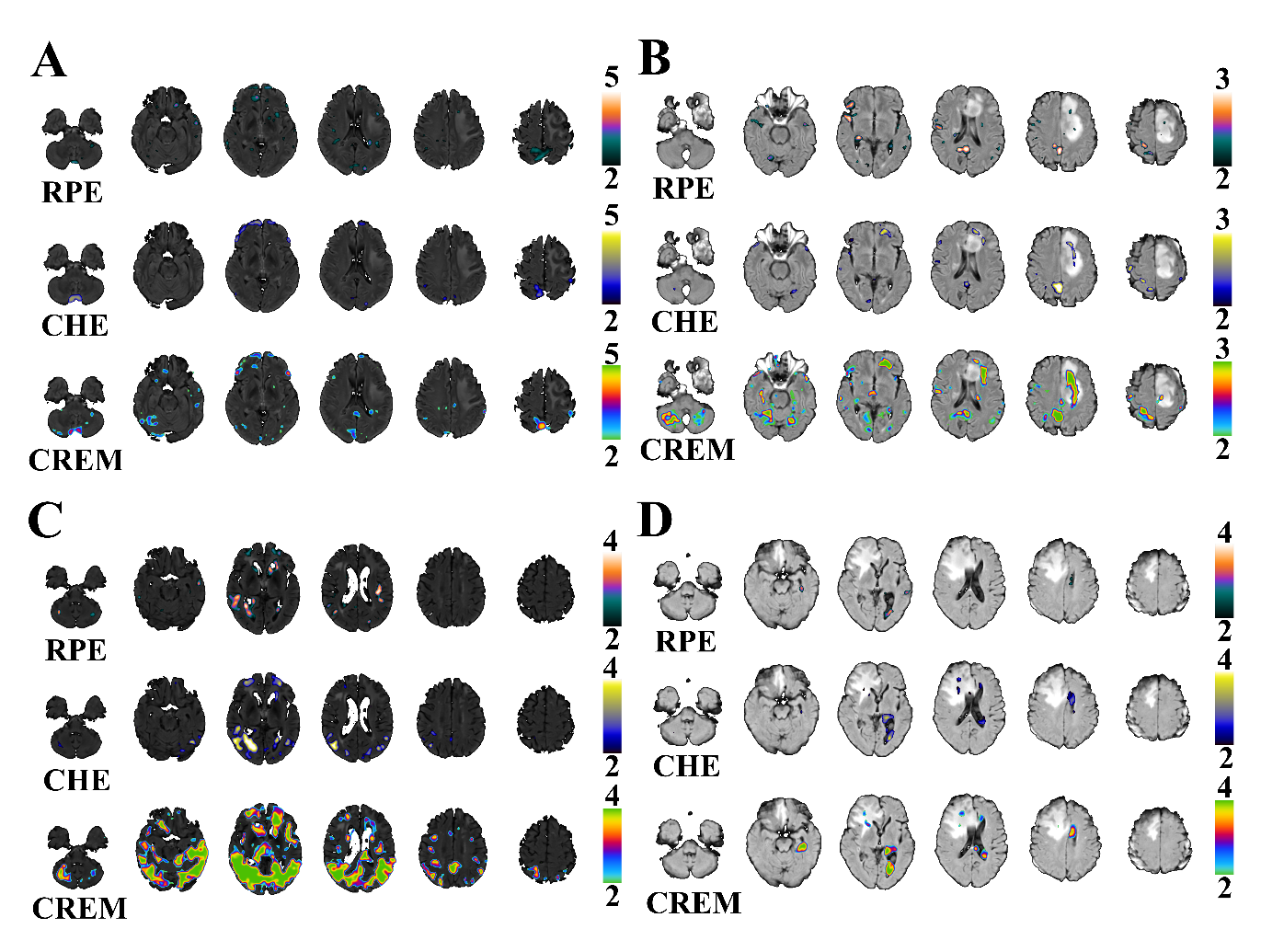
**

**Supplementary Figure 4. The brain areas repeatedly showing increased MREG_BOLD_ amplitudes in serial examination of primary central nervous system lymphoma (PCNSL) patients (*n* = 4).** This figure illustrates cumulative incidence maps from *repeat*ed MREG_BOLD_ scanning in four PCNSL patients (panels A to D). The calculation resembles the above-noted cumulative incidence maps, with the notable difference that these cumulative incidence maps are subject-specific, and not group-specific. In essence, these maps represent the number of scans (2 to 5, see color bars) in an indivudal that demonstrated “markedly increased MREG_BOLD_ amplitudes” or more specifically Z-scores ≥ 3 within the following MREG_BOLD_ amplitudes: respiratory pulse envelope (AF_RPE_), cardiovascular pulse envelope (AF_CHE_), and cardiorespiratory envelope modulation (AF_CREM_).

**Supplementary Tables**

**Supplementary Table 1.** Differences in magnetic resonance encephalography (MREG_BOLD_) amplitudes between primary central nervous system lymphoma (PCNSL) patients (*n* = 30) and control subjects (*n* = 40) after adjusting for covariates.

|  | **Difference after covariate adjustment** | | | |
| --- | --- | --- | --- | --- |
|  | **No covariates** | **Sex** | **Abs** | **Abs+sex** |
| **AF_CREM_** | **GT** | **ns** | **GT** | **ns** |
| **AF_RPE_** | **GT** | **GT** | **GT** | **GT** |
| **AF_CHE_** | **GT** | **GT** | **GT** | **ns** |

Results from voxel-wise nonparametric, threshold-free nonparametric test (*p* ≤ 0.05; False Discovery Rate-corrected, 10 000 permutations). AF_CREM_ = amplitude of cardiorespiratory envelope modulation, AF_CHE_ = amplitude of cardiovascular pulse envelope, AF_RPE_ = amplitude of respiratory pulse envelope. Abs = Absolute head displacement. GT = PCNSL group has greater amplitudes than the control group. Ns = non-significant difference in respective amplitudes between groups.

**Supplementary Table 2.** Amplitudes of physiological fluctuations within anatomical regions of interest

| **Region of Interest** | **Amplitude (x10^5^ a.u)** | | **Significance (*p*-value)** |
| --- | --- | --- | --- |
|  | **Controls (*n* = 40)** | **PCNSL (*n* = 30)** |  |
| **Whole brain** |  |  |  |
| AF_CREM_ | 0.15 [0.14 to 0.17] | 0.18 [0.14 to 0.25] | 0.02 (*) |
| AF_CHE_ | 0.07 [0.06 to 0.07] | 0.07 [0.07 to 0.08] | 0.04 (*) |
| AF_RPE_ | 0.10 [0.09 to 0.12] | 0.13 [0.11 to 0.15] | 0.006 (**) |
| **Lateral Ventricle** |  |  |  |
| AF_CREM_ | 0.67 [0.57 to 1.03] | 1.12 [0.73 to 1.32] | 0.02 (*) |
| AF_CHE_ | 0.33 [0.30 to 0.42] | 0.49 [0.29 to 0.57] | 0.14 (ns) |
| AF_RPE_ | 0.21 [0.17 to 0.24] | 0.29 [0.24 to 0.41] | 0.003 (**) |
| **White Matter** |  |  |  |
| AF_CREM_ | 0.12 [0.10 to 1.34] | 0.14 [0.11 to 0.23] | 0.04 (*) |
| AF_CHE_ | 0.06 [0.06 to 0.07] | 0.07 [0.06 to 0.09] | 0.33 (ns) |
| AF_RPE_ | 0.09 [0.08 to 0.10] | 0.12 [0.10 to 0.14] | 0.01 (*) |
| **Grey Matter** |  |  |  |
| AF_CREM_ | 0.14 [0.12 to 0.16] | 0.16 [0.13 to 0.23] | 0.14 (ns) |
| AF_CHE_ | 0.06 [0.05 to 0.07] | 0.06 [0.06 to 0.08] | 0.16 (ns) |
| AF_RPE_ | 0.10 [0.09 to 0.13] | 0.13 [0.12 to 0.15] | 0.02 (*) |
| **Tumor** |  |  |  |
| AF_CREM_ |  | 0.25 [0.20 to 0.30] | X < 0.0001 (****) |
| AF_CHE_ |  | 0.11 [0.09 to 0.14] | X < 0.0001 (****) |
| AF_RPE_ |  | 0.14 [0.12 to 0.16] | X 0.0018 (**) |
| **Peritumoral Area** |  |  |  |
| AF_CREM_ |  | 0.21 [0.17 to 0.32] | X < 0.0001 (****) |
| AF_CHE_ |  | 0.09 [0.08 to 0.13] | X < 0.0001 (****) |
| AF_RPE_ |  | 0.14 [0.11 to 0.15] | X 0.0099 (**) |
| **Tumor** |  |  |  |
| AF_CREM_ |  | 0.25 [0.20 to 0.30] | Δ < 0.0001 (****) |
| AF_CHE_ |  | 0.11 [0.09 to 0.14] | Δ < 0.0001 (****) |
| AF_RPE_ |  | 0.14 [0.12 to 0.16] | Δ 0.30 (ns) |
| **Peritumoral Area** |  |  |  |
| AF_CREM_ |  | 0.21 [0.17 to 0.32] | Δ 0.0003 (***) |
| AF_CHE_ |  | 0.09 [0.08 to 0.13] | Δ < 0.0001 (****) |
| AF_RPE_ |  | 0.14 [0.11 to 0.15] | Δ 0.86 (ns) |

Note: The physiological pulsation mechanisms as detected with MREG_BOLD_ scanning: CREM (*cardiorespiratory envelope modulation*), CHE (*cardiovascular* *pulse envelope modulation*), RPE (*respiratory pulse envelope modulation*). In PCNSL patients, tumor areas (as defined as macroscopic hyperintense FLAIR areas) have been excluded from white matter and grey matter analysis, and cerebrospinal fluid (CSF) areas have been excluded from peritumoral analysis. Values represent group median and confidence limits of the 95 % Confidence Interval. Statistical *p*-values represent results from exact two-tailed Mann-Whitney U-tests or exact two-tailed Wilcoxon matched pairs signed rank test in the case of matched pairs. Since control subjects do not have comparable tumor areas, we compared (peri)tumoral amplitudes against *control subjects’ whole-brain amplitudes* whereby these p-values are denoted with an X symbol, Similarly, we compared (peri)tumoral PCNSL amplitudes against *PCNSL patients’ whole brain amplitudes* whereby these p-values are denoted with a delta (Δ) symbol.

**Supplementary Table 3**. Significant Spearman correlation coefficients (with the respective p-values in brackets) between region-specific MREG_BOLD_ amplitudes and clinical factors of patients with primary central nervous system lymphoma (PCNSL).

| **Amplitude** | **MSKCC** | **Age** | **FLAIR** | **Maximum Gd+** | **Summated Gd+** | **Multifocality** |
| --- | --- | --- | --- | --- | --- | --- |
| **Whole brain** |  |  |  |  |  |  |
| **CREM** |  |  |  |  |  | 0.41 (0.026) |
| **RPE** |  |  |  |  |  |  |
| **CHE** |  |  |  |  |  | 0.49 (0.006) |
| **Lateral Ventricle** |  |  |  |  |  |  |
| **CREM** |  |  |  |  |  | 0.53 (0.003) |
| **RPE** |  |  |  |  |  | 0.52 (0.003) |
| **CHE** |  |  |  |  |  |  |
| **White Matter** |  |  |  |  |  |  |
| **CREM** |  | 0.43 (0.018) |  |  |  |  |
| **RPE** |  |  |  |  |  |  |
| **CHE** |  | 0.44 (0.014) |  |  |  | 0.41 (0.025) |
| **Grey Matter** |  |  |  |  |  |  |
| **CREM** |  |  |  |  |  |  |
| **RPE** |  |  |  |  |  |  |
| **CHE** |  |  |  |  |  | 0.41 (0.024) |
| **Tumor** |  |  |  |  |  |  |
| **CREM** |  |  |  |  |  | 0.47 (0.009) |
| **RPE** |  |  |  |  |  | 0.39 (0.034) |
| **CHE** |  |  | -0.40 (0.027) | -0.37 (0.045) |  | 0.44 (0.015) |
| **Peritumoral** |  |  |  |  |  |  |
| **CREM** |  | 0.36 (0.048) |  |  |  | 0.38 (0.041) |
| **RPE** |  |  |  |  |  |  |
| **CHE** |  |  |  |  |  |  |

Note: Region-specific MREG_BOLD_ amplitudes correspond to the following pulsation mechanisms: cardiorespiratory envelope modulation (CREM), respiratory pulse envelope modulation (RPE), and cardiovascular pulse envelope modulation (CHE). The region-specific MREG_BOLD_ amplitudes were correlated with the following clinical factors: Memorial Sloan Kettering Cancer Center (MSKCC) scores, age, volume for FLAIR hyperintensity (FLAIR), *maximum* contrast-enhancing tumor diameter (Maximum Gd+), summation of all contrast-enhancing tumor diameters (Summated Gd+), number of contrast-enhancing foci (multifocality).
